# The aryl hydrocarbon receptor (AHR) drives human leukocyte antigen (HLA)-II expression in human melanoma

**DOI:** 10.1186/s13046-026-03673-y

**Published:** 2026-02-20

**Authors:** Yiteng Jin, Wenjin Zheng, Rui Zhang, Sen Hou, Ce Luo, Pengfei Ren, Deng Pan, Chunxiong Luo, Zexian Zeng

**Affiliations:** 1 Center for Quantitative Biology, Academy for Advanced Interdisciplinary Studies, Peking University, Beijing, 100871 China; 2 Department of Obstetrics and Gynecology, Chinese PLA Medical School, Beijing, 100853 China; 3 Peking-Tsinghua Center for Life Sciences, Academy for Advanced Interdisciplinary Studies, Peking University, Beijing, 100871 China; 4 Department of Gastroenterological Surgery, Peking University People’s Hospital, Beijing, 100044 China; 5 Tsinghua-Peking Center for Life Sciences, Tsinghua University, Beijing, 100084 China; 6 Department of Basic Medical Sciences, Tsinghua University, Beijing, 100084 China; 7 The State Key Laboratory for Artificial Microstructures and Mesoscopic Physics, School of Physics, Peking University, Beijing, 100871 China; 8 Zhejiang Key Laboratory of Soft Matter Biomedical Materials, Wenzhou Institute, University of Chinese Academy of Sciences, Wenzhou, 325000 Zhejiang China; 9 Peking University Chengdu Academy for Advanced Interdisciplinary Biotechnologies, Chengdu, 610213 Sichuan China

**Keywords:** HLA-II, CIITA, AHR-ARNT, Tumor immunity, Cancer immunotherapy

## Abstract

**Background:**

Although HLA-II molecules are classically associated with professional antigen-presenting cells, their expression by cancer cells has been recognized for several decades. It has been linked to immune infiltration, responses to immune checkpoint blockade, and clinical outcomes. However, the regulatory mechanisms governing tumor-associated HLA-II expression remain incompletely understood.

**Methods:**

Genome-wide CRISPR-Cas9 screening was employed to identify candidate regulators of HLA-II expression in human melanoma cells. Key candidates were functionally validated through genetic and pharmacological perturbation approaches. Integrated transcriptomic and epigenomic analyses were conducted to characterize regulatory mechanisms. Retrospective clinical analyses were performed using publicly available The Cancer Genome Atlas (TCGA) datasets to assess associations with immune infiltration, immunotherapy response, and survival.

**Results:**

We identified the aryl hydrocarbon receptor (AHR) and its dimerization partner ARNT as critical, FICZ-responsive, positive regulators of HLA-II expression. AHR-ARNT promoted transcription of *CIITA* through direct binding to its promoter II (pII), in the absence of IFN-γ signaling. Clinically, an AHR-ARNT loss-of-function signature correlated with reduced immune infiltration, poorer response to immunotherapy, and inferior survival across cancer types.

**Conclusions:**

These findings reveal a previously unrecognized regulatory axis controlling HLA-II expression on cancer cells, suggesting that targeting the AHR-ARNT pathway may enhance tumor immunogenicity and improve immunotherapy efficacy.

**Supplementary Information:**

The online version contains supplementary material available at 10.1186/s13046-026-03673-y.

## Background

Cancer immunotherapy has significantly reshaped the treatment paradigm for various malignancies by harnessing the immune system’s inherent capacity to recognize and eliminate tumor cells. Among these approaches, immune checkpoint blockade (ICB) has demonstrated the potential for durable responses and prolonged survival in patients [1]. Traditionally, human leukocyte antigen class I (HLA-I) molecules have long been the primary focus, due to their essential role in presenting intracellular antigens to cytotoxic CD8 + T cells, enabling targeted immune surveillance and elimination of malignant cells [2]. Growing evidence highlights a critical yet often overlooked role for HLA class II (HLA-II) molecules in orchestrating effective anti-tumor immunity.

HLA-II molecules present exogenous or processed endogenous antigens to CD4 + helper T cells, which play a central role in coordinating adaptive immune responses, including the priming of CD8 + cytotoxic T cells, activation of B cell-mediated antibody production, and maintenance of long-term immunological memory [3]. Under physiological conditions, HLA-II expression is most prominently observed in professional antigen-presenting cells (pAPCs), such as dendritic cells, macrophages, and B cells, but is also present in a range of non-hematopoietic cell types in specific tissues or inducible contexts, including various epithelial and cancer cell types [3–7]. Within the tumor microenvironment (TME), HLA-II expression on cancer cells can be induced by cytokines such as IFN-γ [3, 8]. Notably, recent large-scale transcriptomic analyses, including data from the Cancer Cell Line Encyclopedia (CCLE) [9] and an independent study of 675 cancer cell lines [10], have revealed constitutive HLA-II expression in multiple human solid tumor cell lines, particularly in melanomas and subsets of lung cancers, even in the absence of external stimulation [11]. While some normal non-hematopoietic tissues, such as skin, breast, lung, and kidney tissues, also express HLA-II under specific conditions [12], the molecular mechanisms driving constitutive HLA-II expression in human non-hematopoietic cancer cells remain poorly understood. Intriguingly, this phenomenon appears to be species-specific: whereas numerous human tumor cell lines robustly express HLA-II, most murine non-hematopoietic cancer models, including commonly used lines such as B16F10 melanoma and MC38 colon carcinoma, exhibit minimal to no endogenous expression of MHC-II counterparts [13].

The expression of HLA-II genes is primarily regulated at the transcriptional level by the HLA class II transactivator (CIITA), a non-DNA-binding coactivator that integrates upstream signaling cues to drive HLA-II gene expression. *CIITA* transcription is governed by multiple distinct promoters, pI, pIII, and pIV, which are preferentially utilized in dendritic cells, B cells, and IFN-γ–stimulated cells, respectively, and are conserved across species [14]. In contrast, promoter II (pII) activity appears to be unique to human cells and has been detected in a limited number of cell types, with its physiological relevance and regulatory function remaining incompletely defined [14,15]. The use of specific *CIITA* promoters is both cell-type-specific and context-dependent, contributing to the heterogeneity of HLA-II expression across immune and non-immune cell populations. Notably, in human melanoma cells, aberrant CIITA transcription can arise from non-canonical promoters such as pIII and pIV, which may account for the observed constitutive HLA-II expression even in the absence of inflammatory stimuli such as IFN-γ [16, 17].

HLA-II expression on cancer cells has been associated with increased immune cell infiltration, enhanced responses to immune checkpoint blockade (ICB), and improved clinical outcomes [11]. In murine models of non-small cell lung cancer (NSCLC), MHC-II expression on cancer cells correlates with higher infiltration and activation of both CD4 + and CD8 + T cells, elevated cytokine production, and enhanced sensitivity to anti-PD-1 therapy [5]. Similarly, in lung adenocarcinoma patients, higher HLA-II expression is linked to increased overall infiltration of CD4 + and CD8 + T cells, as well as closer spatial proximity between immune and cancer cells [4]. In melanoma, HLA-II on cancer cells serves as a predictive biomarker for clinical responses to anti-PD-1 and anti-PD-L1 therapies [18,19]. Comparable associations have also been reported in breast cancers, particularly in triple-negative breast cancer (TNBC) and HER2-negative subtypes, where tumor HLA-II expression correlates with lymphocyte infiltration and therapeutic benefit from anti-PD-1 blockade [20–23]. Collectively, these findings suggest that HLA-II on cancer cells not only reflects an immunologically active tumor microenvironment but may also serve as a functional mediator of therapeutic response.

Despite the strong correlations between tumor-associated HLA-II expression and immunological and clinical outcomes, the molecular mechanisms that regulate HLA-II expression on cancer cells remain largely unexplored. Elucidating these regulatory pathways is essential for developing therapeutic strategies that harness the antigen-presenting capacity of tumor cells to enhance antitumor immunity. While prior studies have identified both transcriptional and post-translational mechanisms by which cancer cells suppress HLA-II expression and evade immune detection [24–27], the molecular pathways that actively promote or sustain basal HLA-II expression on tumor cells are largely unknown.

The aryl hydrocarbon receptor (AHR) is a ligand-activated transcription factor that responds to a broad range of endogenous and exogenous ligands, including tryptophan-derived metabolites such as 6-formylindolo[3,2-b]carbazole (FICZ). Upon ligand binding, AHR heterodimerizes with the aryl hydrocarbon receptor nuclear translocator (ARNT) and regulates gene transcription through AHR response elements (AHREs) [28]. While AHR signaling has been extensively studied in the context of xenobiotic metabolism and immune regulation [29–32], its potential role in controlling basal HLA-II expression on tumor cells has not been explored.

In this study, we aimed to identify regulators of basal HLA-II expression in human melanoma cells. Through genome-wide CRISPR-Cas9 screening combined with functional and mechanistic validation, we identified the AHR and its dimerization partner ARNT as key positive regulators of HLA-II expression on cancer cells. Genetic ablation of either *AHR* or *ARNT* markedly reduced surface HLA-II levels, while ectopic expression of these factors enhanced HLA-II expression in the absence of IFN-γ signaling. Reintroduction of *AHR* or *ARNT* into their respective knockout cells rescued HLA-II expression, confirming their functional necessity. Systematic transcriptomic, chromatin accessibility, and chromatin immunoprecipitation analyses revealed that the AHR-ARNT complex binds directly to the pII promoter of *CIITA* and concurrently enhances the transcriptional activity of the pIII and pIV promoters. Together, these findings uncover a previously unrecognized regulatory axis that could potentially enhance tumor immunogenicity and improve the efficacy of cancer immunotherapy.

## Methods

### Cell lines

A375 (RRID: CVCL_0132), WM115 (RRID: CVCL_0040), SKMEL2 (RRID: CVCL_0069), B16F10 (RRID: CVCL_0159), LLC (RRID: CVCL_4358), MC38 (RRID: CVCL_B288), and HEK293T (RRID: CVCL_0063) cells were cultured in DMEM supplemented with 10% fetal bovine serum (FBS), 100 µg/mL penicillin and 100 U/ml streptomycin at 37 °C in 5% CO_2_.

### Viral packaging

For viral production, 293T cells were plated in 15-cm culture dishes and transfected once they reached approximately 80% confluency, typically 12–18 h later. For lentiviral packaging, transfection mixtures were prepared containing 20 µg of either sgRNA or target plasmid, 13.5 µg of psPAX2 (RRID: Addgene_12260), 6.5 µg of pMD2.G (RRID: Addgene_12259), and 120 µL of PEI (Polysciences #24765-100) in Opti-MEM (Gibco, #11058021). Plasmid DNA and PEI were each pre-incubated in Opti-MEM for 5 min before being combined. The resulting transfection complexes were allowed to incubate at room temperature for 30 min before being added to the cells. Six hours after transfection, the medium was replaced with fresh growth medium. Viral supernatants were collected 48 h later, passed through 0.45 μm filters, aliquoted into 1 mL portions, and stored at − 80 °C for future use.

### Genome-wide CRISPR screening for HLA-II

A375-Cas9 and WM115-Cas9 were generated by transfection with lentivirus encoding Cas9-Blast (Addgene #52962) and selected with 6 µg/mL and 12 µg/mL blasticidin (InvivoGen #ant-bl-05) separately for 10 days. These two cell lines were independently transduced with Brunello lentivirus library (Addgene #73178) at an infection rate of around 20%. sgRNA coverage was maintained throughout the experiments at > 500 copies of each sgRNA (~ 40 million cells for the 77,441 sgRNAs). After 48 h of transfection, cells that had been transduced were selected using 0.7 µg/ml (A375) or 0.6 µg/ml (WM115) of puromycin (InvivoGen #ant-pr-1) for 3 days. 10 days after viral transduction, the selected WM115 cells were treated with 100 ng/mL IFN-γ (Novoprotein #C014). After 13 days of transduction, the cells were collected and divided into an experimental group and a control group, each with three replicates. Each replicate contained ~ 56 million cells (~ 700× in sgRNA coverage). The experimental groups were stained with the FITC-conjugated anti-HLA-DR antibody LN3 (Biolegend #327006), incubated on ice and protected from light for 20 min, washed with PBS, and resuspended with 2 mL PBS plus 5% fetal bovine serum prior to sorting for the lowest and highest ~ 10% populations, yielding ~ 2–4 million cells for each final population. The genomic DNA of the sorted samples and the control group was extracted using the NucleoSpin Blood XL kit (MACHEREY-NAGEL #740950.50), following the manufacturer’s instructions. Amplification of the sgRNA cassettes by PCR was performed according to the broad GPP protocol (https://portals.broadinstitute.org/gpp/public/resources/protocols).

### Data analysis for CRISPR screens

MaGeCK (Model-based Analysis of Genome-wide CRISPR-Cas9 Knockout) was employed to process and analyze the CRISPR screen data [33]. FASTQ reads from the CRISPR screen trimmed and mapped to the corresponding library using MAGeCK “count” function to quantify sgRNA read counts. The MAGeCK “test” module was then used to calculate log2 fold changes and p-values of both the sgRNAs and genes. Custom R (v4.4.1) scripts were used to visualize the data.

### Generation of KO cell lines

The sgRNA sequences used to generate KO cell lines were listed in Additional file 1. Each sgRNA was cloned into lentiGuide-Puro backbone (Addgene #52963), with successful sgRNA insertion confirmed by Sanger sequencing. Lentiviral particles were produced as described above. The virus was used to infect the A375, WM115 and SKMEL2 cells. Following 72-hour infection, puromycin (0.7 µg/mL for A375, 0.6 µg/mL for WM115, and 0.5 µg/mL for SKMEL2) was added to the culture for selection of stable KO cell lines.

### Generation of overexpression cell lines

A375 cells were lysed using TRIzol (Invitrogen, #15596026), and cDNA was synthesized from total mRNA with Evo M-MLV Plus 1st Strand cDNA Synthesis Kit (AGBio #AG11615). The CDS sequences of *AHR* and *ARNT* were then amplified using corresponding primers and cloned into pHAGE vectors with RFP reporter. Lentiviral particles were produced as described above. The virus was used to infect the A375, WM115 and SKMEL2 cells. Following 5 days of viral transduction, the transduced cells were FACS sorted according to RFP signaling.

### In vitro AHR agonist and antagonist treatment experiments

Tumor cells (0.1 million) were plated in 12-well plates per well and incubated for 24 h, 48 h, or 72 h with complete medium containing 0.1% DMSO, 1 µM FICZ, 2.5 µM GNF351, or 100 ng/mL IFN-γ. Cells were stained with APC-conjugated anti-HLA-DR, DP, DQ antibody Tü39 (Biolegend #361714) in FACS buffer (PBS supplemented with 5% FBS), incubated on ice and protected from light for 20 min, washed with PBS buffer, and then stained with DAPI in PBS to distinguish live and dead cells and analyzed by Beckman CytoFLEX S.

### Western blot

Whole-cell lysates were solubilized in cell lysis buffer (Beyotime #P0013). Protein concentrations were determined using the BCA Protein Assay Kit (Solarbio #PC0020), and 20 µg of total protein was loaded per lane onto SDS-PAGE gels. Proteins were transferred to Immobilon PVDF membranes (Millipore). Membranes were blocked in TBST containing 5% non-fat milk for 1 h at room temperature, then incubated overnight at 4 °C with primary antibodies diluted 1:1000 in primary antibody dilution buffer (Solarbio, #A1810). The following primary antibodies were used: AHR Rabbit mAb (clone D5S6H, CST #83200, RRID: AB_2800011), ARNT Rabbit mAb (clone D28F3, CST #5537, RRID: AB_10694232), HLA-DRA polyclonal Rabbit Ab (Boster #A01195), β-Tubulin Mouse mAb (clone C66, Abmart #M20005, RRID: AB_2920648), and β-Actin Rabbit mAb (clone 13E5, CST #4970, RRID: AB_2223172). After washing, membranes were incubated with HRP-conjugated polyclonal secondary antibody anti-rabbit IgG (CST #7074, RRID: AB_2099233, 1:10000 dilution) or anti-mouse IgG (CST #7076, RRID: AB_330924, 1:10000 dilution) for 1 h at room temperature. Blots were visualized using M5 HiPer ECL Western HRP Substrate (Mei5 Biotechnology #MF074-01), and chemiluminescence signals were captured using a ChemiDoc™ Imaging System (Bio-Rad Laboratories).

### Flow cytometry

Adherent cells were dissociated into single-cell suspensions and stained with appropriate fluorochrome-conjugated antibodies. Flow cytometric analysis was performed on a Beckman CytoFLEX S flow cytometer, and cell sorting was conducted using a BD Aria Fusion cell sorter. Data were analyzed with FlowJo software (BD Biosciences, RRID: SCR_008520). All flow cytometry antibodies were purchased from BioLegend, including: FITC anti-human HLA-DR Antibody (clone LN3, Biolegend #327005, RRID: AB_893577), APC anti-human HLA-DR, DP, DQ Antibody (clone Tü39, Biolegend #361714, RRID: AB_2750316), PE anti-human HLA-A, B, C Antibody (clone W6/32, Biolegend #311406, RRID: AB_314875), APC anti-human HLA-A, B, C Antibody (clone W6/32, Biolegend #311410, RRID: AB_314879), FITC anti-mouse I-A/I-E Antibody (clone M5/114.15.2, Biolegend #107605, RRID AB_313320), and FITC Mouse IgG2b, κ Isotype Ctrl Antibody (clone 27–35, Biolegend #402207, RRID: AB_3097051). The allele specificities of the antibodies used to label MHC molecules are as follows: LN3 reacts with the conserved structure of the HLA-DR protein; Tü39 reacts with a shared epitope of HLA-DR, HLA-DP, and HLA-DQ; W6/32 reacts with a pan–HLA-I combinatorial determinant including the class I heavy chain and β2-microglobulin residues; M5/114.15.2 reacts with a polymorphic determinant shared by the I-A^b, d, q^ and I-E^d, k^ MHC class II alloantigens from mice carrying H-2^p, r, q, b, d, u^ haplotypes.

### RNA-seq

For RNA-seq analysis, we included A375 and WM115 cells expressing non-targeting control gRNA, AHR-targeting gRNA (AHR KO), or ARNT-targeting gRNA (ARNT KO); A375 and WM115 cells treated with 0.1% DMSO or 1 µM FICZ for 72 h; and SKMEL2 cells transduced with vector control, AHR overexpression (AHR OE), or ARNT overexpression (ARNT OE) constructs. All cells were cultured in 6-well plates in triplicate. A minimum of 1 × 10^6^ cells per sample were collected across all groups. The cells were washed with PBS and lysed using TRIzol (Invitrogen #15596026). Using 1 µg of total RNA, RNA libraries for RNA-seq were prepared using VAHTS Universal V6 RNA-Seq Library Prep Kit for Illumina (Vazyme #NR604-01/02) according to the manufacturer’s protocols, followed by Illumina sequencing. The reads were aligned to the human reference genome hg38 using STAR (RRID: SCR_004463). Feature count was used to map aligned reads to genes and generate a gene count matrix.

### ATAC-seq

For ATAC-seq analysis, we included A375 and WM115 cells expressing non-targeting control gRNA, AHR-targeting gRNA (AHR KO), or ARNT-targeting gRNA (ARNT KO); A375 and WM115 cells treated with 0.1% DMSO or 1 µM FICZ for 72 h; and SKMEL2 cells transduced with vector control, AHR overexpression (AHR OE), or ARNT overexpression (ARNT OE) constructs. All cells were cultured in 6-well plates in triplicate. For each sample, 1 million cells were collected, washed with PBS, resuspended with 20 µL PBS, and lysed with lysis buffer (10 mM Tris-HCl, pH 7.4, 10 mM NaCl, 3 mM MgCl_2_, 0.5% NP-40). The ATAC library for each sample was then prepared using TruePrep DNA Library Prep Kit V2 for Illumina (Vazyme #TD501) with TruePrep Index Kit V2 for Illumina (Vazyme #TD202) according to the manufacturer’s protocols, followed by Illumina sequencing. The FASTQ reads were trimmed and aligned to the hg38 reference genome using Bowtie 2 (RRID: SCR_016368), followed by proper filtering using the SAMtools (RRID: SCR_002105) pipeline. BigWig coverage tracks were generated using deepTools (RRID: SCR_016366) bamCoverage (v3.5.3) with 10 bp bin size and RPGC normalization (effective genome size: 2,862,010,428). chrX and chrM were excluded from normalization, and reads were extended to the estimated fragment size.

### ChIP-seq

For ChIP-seq analysis, A375 cells, WM115 cells treated with 1 µM FICZ for 72 h, and SKMEL2 cells expressing 3×HA-RFP, 3×HA-AHR-RFP, or 3×HA-ARNT-RFP were used. All cells were cultured in 15 cm dishes separately. For each ChIP-seq sample, a total of 4 × 10⁷ cells were harvested and resuspended in 2 mL serum-free DMEM prior to cross-linking. Specifically, for A375 cells and WM115 cells treated with 1 µM FICZ for 72 h, 1.2 × 10⁸ cells were equally divided into three aliquots (4 × 10⁷ cells each) for ChIP with control IgG (clone DA1E, CST #3900, RRID: AB_1550038), anti-AHR (clone D5S6H, CST #83200, RRID: AB_2800011), or anti-ARNT (clone D28F3, CST #5537, RRID: AB_10694232) antibodies, respectively. For SKMEL2 cells expressing 3×HA-RFP, 3×HA-AHR-RFP, or 3×HA-ARNT-RFP, 4 × 10⁷ cells per group were subjected to ChIP using an anti-HA tag monoclonal antibody (clone 1F5C6, Proteintech #66006-2-Ig, RRID: AB_2881490).

For cross-linking, 1% formaldehyde solution (prepared freshly) was added, and cells were incubated at room temperature (RT) for 8 min. Cross-linking was quenched by adding 0.125 M glycine and incubating at RT for 5 min. Cells were then washed once with ice-cold PBS. After fixation, pellets were flash frozen and stored at -80 °C or processed immediately for sonication.

Each pellet was lysed in lysis buffer (0.1% SDS, 1% Triton X-100, 0.1% Na-Deoxycholate, 0.25% sarcosyl, 50 mM HEPES-KOH, pH 7.5, 1 mM EDTA, 140 mM NaCl, 1× protease inhibitor cocktail). Samples were incubated on ice for 10–15 min, then aliquoted into thin-walled 0.5 mL PCR tubes. Sonication was performed using a Qsonica Q800R (50% amplitude, 30 s on / 30 s off, for 20 cycles) to achieve a mean DNA fragment size of 300 bp. Post-sonication, samples were centrifuged at 12,000×g for 5 min at 4 °C, and the supernatant was collected and kept on ice or stored at − 80 °C. Samples were diluted 1:10 in dilution buffer (1% Triton X-100, 140 mM NaCl, 50 mM HEPES-KOH, pH 7.5, and 1 mM EDTA) supplemented with 5 M NaCl to reach 150 mM NaCl, and incubated with the indicated antibodies and Protein A/G magnetic beads (Invitrogen) at 4 °C for 3 h with rotation.

After washing seven times with high salt buffer (0.1% SDS,1%Triton X-100, 20 mM Tris pH 7.9, 2 mM EDTA, 500 mM NaCl) and twice with TE buffer (10 mM Tris, 1 mM EDTA, pH 8.0), samples were eluted from the beads for 15 min at 65 °C in elution buffer (1% SDS, 200 mM NaCl). Eluates were treated with RNase A at 37 °C for 30 min and reverse cross-linked overnight by heating at 65 °C with Proteinase K. Samples were extracted with an equal volume of phenol: chloroform, vortexed, centrifuged at 12,000×g for 10 min. The aqueous phase (~ 400 µL) was transferred to a new tube and mixed with 1.6 µL GlycoBlue (250×), 40 µL 3 M sodium acetate, pH 5.2, and 800 µL ice-cold ethanol. DNA was precipitated at − 20 °C overnight. Samples were centrifuged at 12,000×g for 15 min at 4 °C, washed twice with 75% ethanol, and resuspended in 20 µL nuclease-free water. The ChIP library for each sample was then prepared using VAHTS Universal Pro DNA Library Prep Kit for Illumina (Vazyme #ND608) with VAHTS Multiplex Oligos Set 4 for Illumina (Vazyme #N321) and VAHTS DNA Clean Beads (Vazyme #N411) according to the manufacturer’s protocols, followed by Illumina sequencing. The FASTQ reads were trimmed and aligned to the hg38 reference genome using Bowtie 2 (RRID: SCR_016368), followed by proper filtering using the SAMtools (RRID: SCR_002105) pipeline. BigWig coverage tracks were generated using deepTools (RRID: SCR_016366) bamCoverage (v3.5.3) with 10 bp bin size and RPGC normalization (effective genome size: 2,862,010,428). chrX and chrM were excluded from normalization, and reads were extended to the estimated fragment size. MACS2 (RRID: SCR_013291) was used to call peaks and MEME-ChIP (SCR_001783) was used for motif enrichment analysis.

### ChIP-qPCR

ChIP-qPCR analysis was conducted using ChIP DNA to detect whether AHR and ARNT protein binds to the AHRE region of the promoter pII of the *CIITA* gene. qPCR amplification was performed in technical triplicates using iTaq Universal SYBR^®^ Green Supermix (BIO-RAD #1725121) on a Bio-Rad CFX96 Touch Real-Time PCR Detection System. Fold enrichment between the control and overexpression groups was calculated. The primer sequences used in ChIP-qPCR were listed in Additional file 2.

### Dual luciferase assay

The pGL4.10-pII-luc2 plasmid, in which the transcription of firefly luciferase was driven by the promoter pII of the *CIITA* gene, was constructed. This plasmid was co-transfected into 293T cells with the pGL4.74-TK-hRluc plasmid and one of the following: the AHR-overexpression plasmid, the ARNT-overexpression plasmid, the control plasmid, or H_2_O, for the dual-luciferase reporter assay.

### RT-qPCR

A375, WM115, and SKMEL2 cells pre-treated with 0.1% DMSO, 1 µM FICZ, 2.5 µM GNF351, or 100 ng/mL IFN-γ for 72 h, were lysed, and total RNA was extracted using RNAsimple Total RNA kit (TIANGEN, Cat# DP419) according to the manufacturer’s instructions. Subsequent reverse transcription was performed with 4 µg of total RNA as template using Evo M-MLV Plus 1st Strand cDNA Synthesis Kit (AGBio #AG11615). qPCR amplification was performed in technical triplicates using iTaq Universal SYBR^®^ Green Supermix (BIO-RAD #1725121) on a Bio-Rad CFX96 Touch Real-Time PCR Detection System. *CIITA* products were amplified using the following primers specific for, respectively, CIITA-pI, CIITA-pII, CIITA-pIII, CIITA-pIV, and CIITA-exon 2 (reverse) (see Additional file 3). As a positive control, we amplified *CYP1A1* products in all samples. The relative expression levels of target genes were normalized to the endogenous reference gene *GAPDH* using the comparative Ct (ΔΔCt) method, and log2 fold changes between the drug-treated and DMSO control groups were calculated.

### Analysis of the cancer genome atlas (TCGA) cohorts

Transcriptome data and clinical data were obtained from the TCGA Data Portal (https://www.cancer.gov/tcga). We chose all 33 cancer types with transcriptome data available for cancer samples. Only those samples in the clinical category of “primary tumor” and “metastatic” were used for this study.

### Statistical analysis

Statistical analyses were performed via GraphPad Prism 9 software (RRID: SCR_002798), applying unpaired Student’s t-test, one-way ANOVA, or two-way ANOVA test as indicated (*p < 0.05, **p < 0.01, ***p < 0.001, ****p < 0.0001). Group sizes for in vitro experiments were set based on prior experience with the experimental variability.

### Differential gene expression (DEG) analyses

For bulk RNA-seq data, the R package DESeq2 (v1.34.0) was used to identify differentially expressed genes between *AHR* or *ARNT* KO versus non-targeting control A375 cells, and FICZ-treated versus DMSO control WM115 cells. Genes with Benjamini–Hochberg-adjusted p value < 0.0001 (for A375 cells) or 0.01 (for WM115 cells) and the absolute log_2_(fold change) > 0.5 were considered differentially expressed genes.

### Kyoto encyclopedia of genes and genomes (KEGG) pathway enrichment analyses

KEGG pathway enrichment analysis was conducted on differentially regulated genes using the enrichKEGG function of the R package clusterProfiler (v4.12.6).

### *AHR-ARNT*-KO signature

To study the clinical relevance of AHR-ARNT, we established an *AHR-ARNT*-KO signature comprising 300 genes (199 upregulated and 101 downregulated) by identifying genes that were differentially expressed (adjusted *p* < 0.0001, |log_2_ fold change| > 0.5) in both *AHR* KO and *ARNT* KO compared with non-targeting controls in A375 cells. For each gene, a weight equal to the mean DESeq2 Wald statistic across the two comparisons was assigned. These weights were than normalized to the − 1 to 1 range by the formula $\:{k}_{i}={w}_{i}/\underset{j}{\mathrm{m}\mathrm{a}\mathrm{x}}\left(\left|{w}_{j}\right|\right)$, where $\:{k}_{i}$ indicates the normalized weight of the $\:i$-th gene, and $\:{w}_{i}$ is its mean Wald statistic. All genes in the *AHR-ARNT*-KO signature and their normalized weights were listed in Additional file 4.

For biological annotation of the signature genes, they were first assigned to KEGG pathways based on enrichment analysis described above, with pathways ranked by adjusted p value. For each gene, the highest-ranked pathway in which it first appeared was used as its annotation, yielding annotations for 64 genes. The remaining genes were subsequently annotated using Gene Ontology Biological Process (GO_BP) enrichment following the same procedure, resulting in annotations for an additional 145 genes. Genes not captured by either KEGG or GO_BP analyses (91 genes) were classified as “Other / poorly characterized” (see Additional file 4).

For each input expression profile, we computed an *AHR-ARNT*-KO signature score to estimate the *AHR-ARNT*-KO level by calculating the weighted sum expression of the signature genes following the equation $\:S={\sum\:}_{i=1}^{n}\left({k}_{i}\times\:{X}_{i}\right)$, where $\:S$ denotes the signature score, and $\:{X}_{i}$ denotes the expression level of the $\:i$-th gene. Finally, we evaluated the association of *AHR-ARNT* deficiency with immune infiltration, response to ICB, and patient outcome.

## Results

### Genome-wide CRISPR screens reveal AHR and ARNT as regulators of HLA-DR expression on cancer cells

**Fig. 1:**
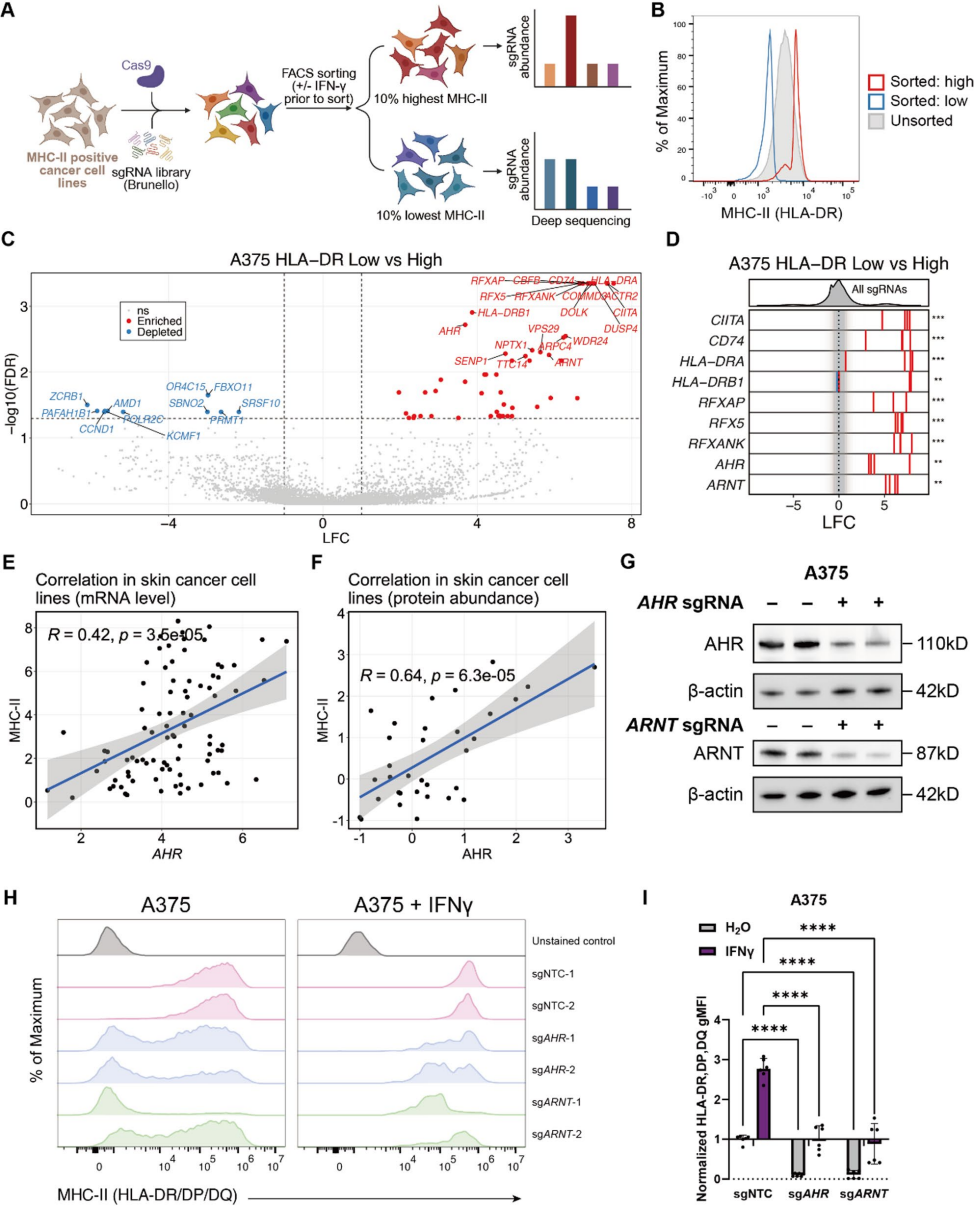
Genome-wide CRISPR screen identifies *AHR* and *ARNT* as positive regulators of HLA-II expression. **A** Schematic overview of the genome-wide CRISPR-Cas9 screen in human melanoma cells to identify regulators of HLA-II surface protein level. **B **Representative flow cytometry histograms from three independent experiments showing sorting of HLA-DRlow and HLA-DRhigh cell populations. **C **Scatterplot of sgRNAs enriched (red) or depleted (blue) in the HLA-DRlow population compared to the HLA-DRhigh population. The top 20 sgRNAs were highlighted. **D **Log2 fold change values of individual sgRNAs plotted in (**C**), showing the effect of gene perturbations on HLA-DR expression. **E**-**F **Scatterplot showing the Pearson correlation between AHR expression and HLA-II expression across skin cancer cell lines in the Cancer Cell Line Encyclopedia (CCLE) at the mRNA level (**E**) and protein level (**F**). **G** Western blot analysis confirming loss of AHR and ARNT protein in A375-Cas9 cells following CRISPR-mediated knockout. Protein levels of AHR and ARNT were assessed using anti-AHR (clone D5S6H) and anti-ARNT (clone D28F3), respectively, with β-actin detected using an anti–β- actin antibody (clone 13E5) as a loading control. **H**-**I** Flow cytometry analysis of surface pan–HLA-II (HLA-DR, DP, DQ) expression in A375-Cas9 cells transduced with sgRNAs targeting AHR, ARNT, or control sgRNAs, with or without IFN-γ treatment (100 ng/mL, 72 h). Representative histograms from three independent experiments show pan–HLA-II expression (**H**). The geometric MFI (gMFI) was quantified across three independent experiments, normalized to the NTC group without IFN-γ treatment, and presented as fold change (**I**). For panel I, data from the two sgRNAs within each group were pooled. Surface HLA-DR expression was detected using FITC anti-human HLA-DR Antibody (clone LN3) (**B**). Surface pan–HLA-II expression was detected using APC anti-human HLA-DR, DP, DQ Antibody (clone Tü39) (**H**-**I**). Data are represented as mean ± SD (**I**). Statistical analysis by two-way ANOVA (**I**); *p < 0.05, **p < 0.01, ***p < 0.001, ****p < 0.0001

To systematically identify genes that regulate HLA-II expression on cancer cells, we conducted genome-wide CRISPR-Cas9 loss-of-function screens in two melanoma cell lines, A375 and WM115, both of which exhibit constitutive surface expression of HLA-DR (Supplementary Fig. 1A). Cas9-expressing derivatives of these cell lines were transduced with the Brunello genome-wide gRNA library [34] (Fig. 1A). Given the intermediate basal level of HLA-DR surface expression in WM115 cells, we pre-treated them with IFN-γ for 72 h prior to screening to enhance the dynamic range for detection. Using flow cytometry and an HLA-DR-specific antibody, we isolated the top and bottom 10% of cells based on surface HLA-DR expression and subsequently quantified sgRNA abundance via deep sequencing (Fig. 1B and Supplementary Fig. 1B).

The screen successfully recovered nearly all known regulators of HLA-II expression and antigen presentation. Genes encoding HLA-II structural components (e.g., *HLA-DRA*, *HLA-DRB1*), the invariant chain (*CD74*), the HLA-II master regulator *CIITA*, and the RFX family transcriptional co-activators (*RFXANK*, *RFX5*, *RFXAP*) were all significantly enriched in the HLA-DR^low^ populations (Fig. 1C-D and Supplementary Fig. 1C-F). In the IFN-γ-treated WM115 screen, multiple components of the IFN-γ signaling pathway, including *IFNGR1*, *IFNGR2*, *JAK1*, *JAK2*, and *STAT1*, also showed strong enrichment in the HLA-DR^low^ population (Supplementary Fig. 1C-D and F), further validating the screening accuracy.

In addition to these established regulators, we identified several candidate genes not previously implicated in HLA-II expression. Among the top-ranked hits, *AHR* was enriched as the only receptor, other than IFN-γ receptors, consistently enriched in the HLA-DR^low^ populations of both A375 and WM115 cells (Fig. 1C-D and Supplementary Fig. 1C-F). Notably, its dimerization partner *ARNT* was also among the top hits in the A375 screen. The co-enrichment of *AHR* and *ARNT* strongly suggested a critical role for the AHR-ARNT complex in promoting HLA-II expression in melanoma cells. Supporting this hypothesis, integrative analysis of the CCLE transcriptomic and proteomic datasets revealed statistically significant, but moderate positive correlations between *AHR* expression and HLA-II expression across multiple cancer types, especially in skin cancers (Fig. 1E-F and Supplementary Fig. 1G-H).

### The AHR-ARNT complex is essential for basal HLA-II expression in human melanoma cells

To validate the role of AHR and ARNT in regulating basal HLA-II expression on cancer cells, we generated *AHR* and *ARNT* knockout (KO) A375 cell lines using CRISPR-Cas9 (Fig. 1G). Notably, residual AHR and ARNT expression were detected after CRISPR knockout, consistent with heterogeneous CRISPR-Cas9 editing efficiency, whereby only a fraction of cells underwent complete gene disruption while others retained partial or intact expression. Loss of either gene resulted in a marked repression in surface HLA-DR expression (Supplementary Fig. 1I-J). Importantly, IFN-γ treatment partially restored HLA-DR expression in both KO lines, although not to the level observed in the untreated control group (Supplementary Fig. 1I-J), indicating that AHR-ARNT deficiency attenuates both basal and IFN-γ–induced HLA-DR expression. Given the high basal expression of pan–HLA-II (HLA-DR, DP, DQ) in A375 cells (Supplementary Fig. 1K), we further assessed its levels and observed a concordant suppression after *AHR* or *ARNT* knockout, which was similarly partially rescued by IFN-γ treatment (Fig. 1H-I and Supplementary Fig. 1L). These findings were further supported by experiments in WM115 and SKMEL2 cells, where AHR or ARNT deletion produced comparable downregulation of surface HLA-II expression (Supplementary Fig. 1L), reinforcing the functional relevance of this regulatory axis.

**Fig. 2:**
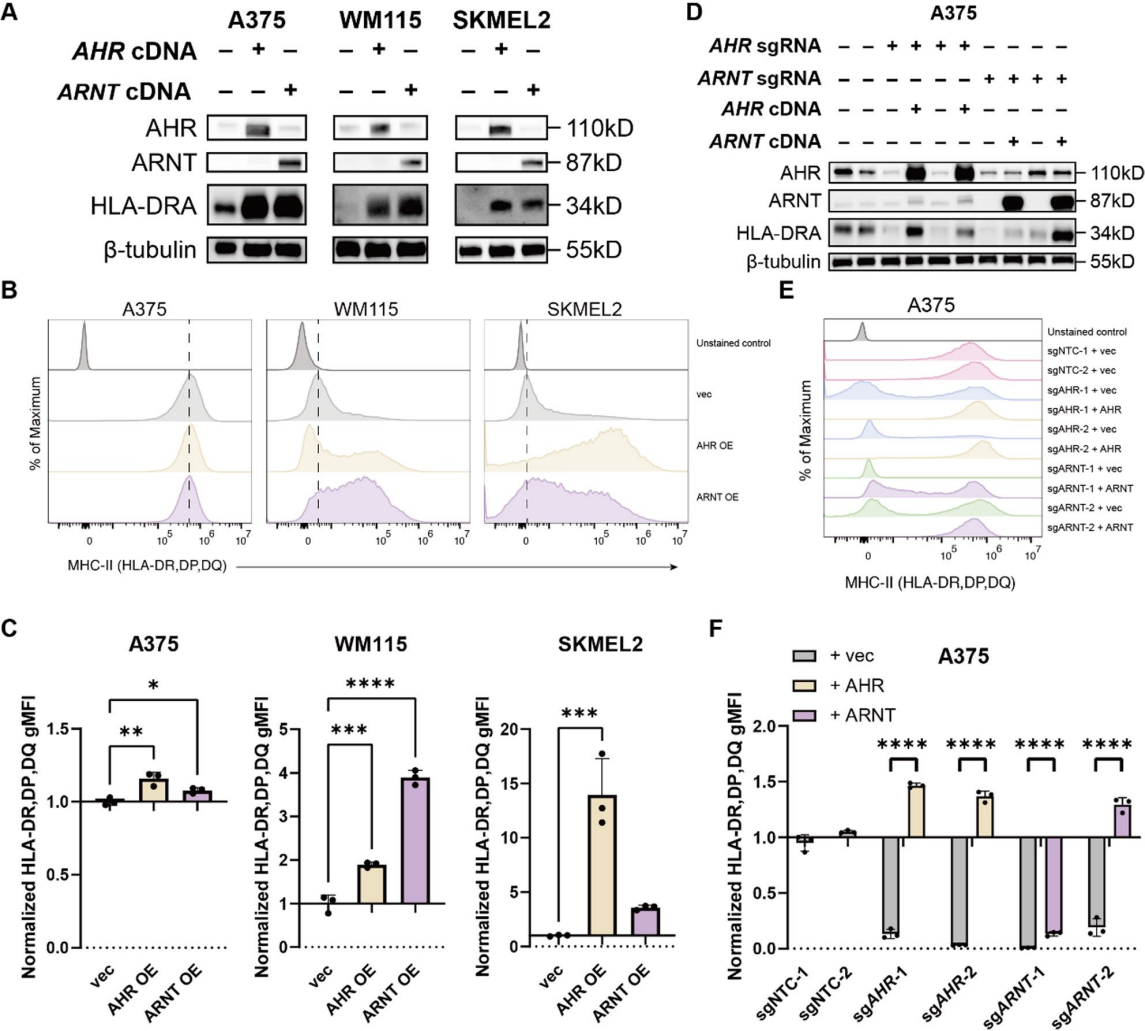
AHR and ARNT regulate basal HLA-II expression on cancer cells. **A **Western blot analysis confirming AHR or ARNT overexpression (OE) in A375, WM115, and SKMEL2 melanoma cell lines. Protein levels of AHR, ARNT, and HLA-DRA were assessed using anti-AHR (clone D5S6H), anti-ARNT (clone D28F3), and polyclonal anti–HLA-DRA antibodies, respectively, with β-tubulin detected using an anti–β-tubulin antibody (clone C66) as a loading control. **B-C** Flow cytometry analysis of surface pan–HLA-II expression in A375, WM115, and SKMEL2 cells overexpressing AHR or ARNT. Representative histograms from three independent experiments show pan–HLA-II expression, with dashed lines indicating the median of the empty vector control group (**B**). The gMFI was quantified across three independent experiments, normalized to the empty vector control group, and presented as fold change (**C**). **D **Western blot analysis of AHR, ARNT, and HLA-DRA protein levels in AHR or ARNT reconstituted A375 cells generated by reintroducing AHR or ARNT into respective KO cells. Protein levels of AHR, ARNT, and HLA-DRA were assessed using anti-AHR (clone D5S6H), anti-ARNT (clone D28F3), and polyclonal anti–HLA-DRA antibodies, respectively, with β-tubulin detected using an anti–β-tubulin antibody (clone C66) as a loading control. **E**-**F** Flow cytometry analysis of surface pan–HLA-II expression in AHR- or ARNT-reconstituted A375 cells. Representative histograms from three independent experiments show pan–HLA-II expression (**E**). The gMFI was quantified across three independent experiments, normalized to the NTC group, and presented as fold change (**F**). Surface pan–HLA-II expression was detected using APC anti-human HLA-DR, DP, DQ Antibody (clone Tü39) (**B**-**C** and **E**-**F**). Data are represented as mean ± SD (**C** and **F**). Statistical analysis by one-way ANOVA (**C**) and unpaired Student’s t-test (**F**); *p < 0.05, **p < 0.01, ***p < 0.001, ****p < 0.0001. vec, empty vector control

To test whether AHR and ARNT are not only necessary but also sufficient to drive HLA-II expression, we overexpressed each factor individually in three human melanoma cell lines, A375, WM115, and SKMEL2, the latter two displaying low and undetectable basal HLA-II expression, respectively (Fig. 2A). Overexpression of either AHR or ARNT led to significant upregulation of HLA-II surface expression in WM115 and SKMEL2 cells, whereas only minimal increases were observed in A375 cells, suggesting that A375 cells likely exhibit saturation of endogenous AHR-ARNT activity (Fig. 2B-C). Notably, this upregulation occurred in the absence of IFN-γ stimulation (Fig. 2B-C), reinforcing that the AHR-ARNT complex is capable of inducing HLA-II expression without canonical inflammatory cues. To confirm the specificity of this regulatory effect, we reintroduced *AHR* and *ARNT* cDNAs, engineered to include three nonconsecutive synonymous mutations in the corresponding sgRNAs targeting sequence and the adjacent PAM sequence, into their respective knockout A375 cells (Supplementary Fig. 2). Re-expression of AHR and ARNT fully rescued HLA-II expression to levels comparable to non-targeting control cells (Fig. 2D-F), again in the absence of IFN-γ. Interestingly, this regulatory mechanism appears to be species-specific. Unlike the human cell lines, the murine melanoma cell line B16F10, colon carcinoma cell line MC38, and lung carcinoma cell line LLC did not express MHC-II at baseline (Supplementary Fig. 3A). Furthermore, overexpression of AHR and ARNT in these mouse lines failed to upregulate MHC-II expression (Supplementary Fig. 3A). These results confirm the functional requirement and sufficiency of the AHR-ARNT complex in promoting basal HLA-II expression on human cancer cells.

### Ligand-dependent AHR activation induces surface HLA-II expression in human melanoma cells

**Fig. 3:**
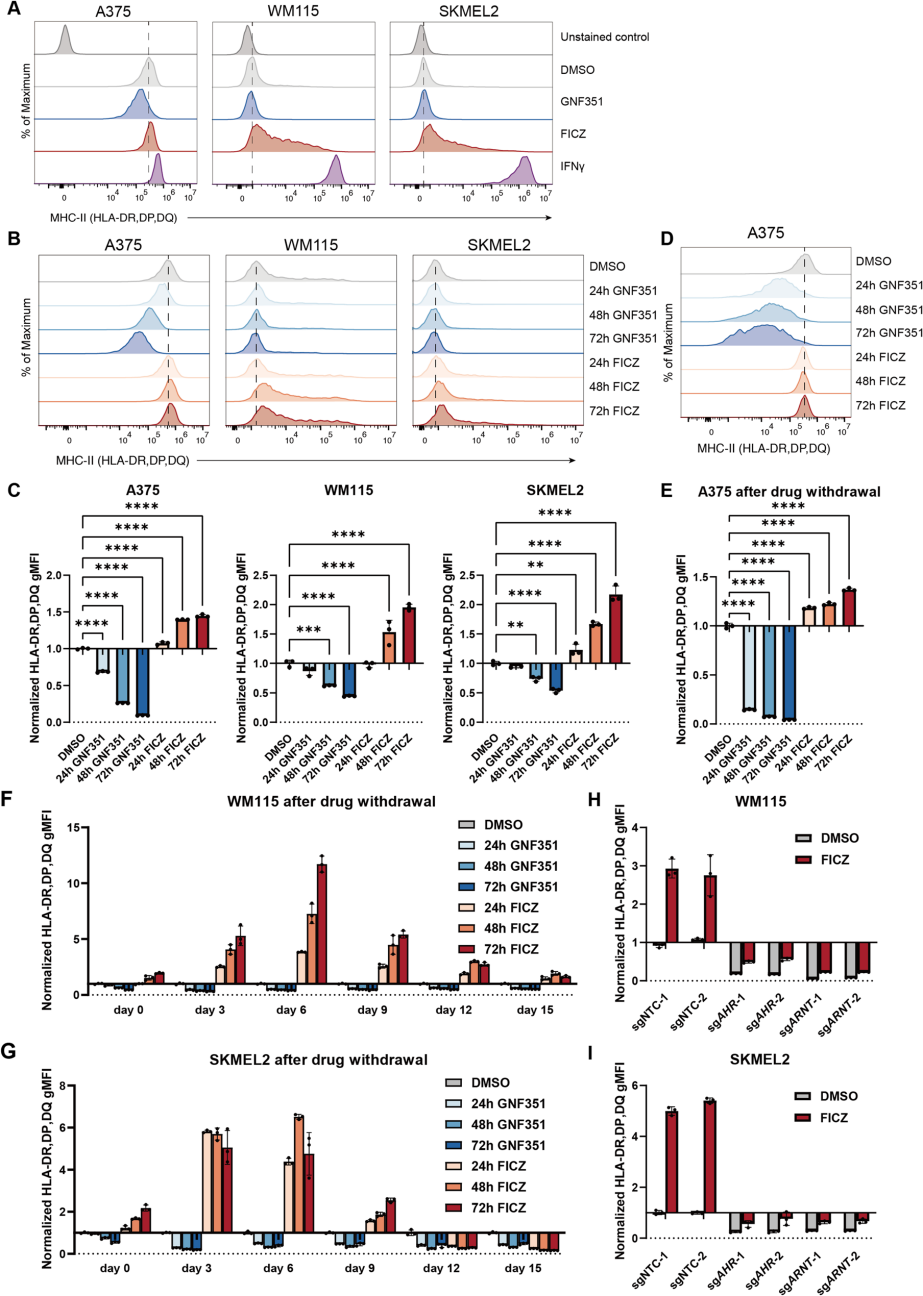
Ligand-mediated activation of AHR regulates HLA-II expression on cancer cells. **A **Flow cytometry analysis of surface pan–HLA-II expression in A375, WM115, and SKMEL2 melanoma cells following 72-hour treatment with 2.5 μM GNF351 (AHR antagonist), 1 μM FICZ (AHR agonist), 100 ng/mL IFN-γ, or 0.1% DMSO (vehicle control). **B**-**C **Time-course analysis of pan–HLA-II surface expression in A375, WM115, and SKMEL2 cells following treatment with GNF351 (2.5 μM) or FICZ (1 μM) for 24, 48, or 72 hours. **D**-**E **pan–HLA-II expression in A375 cells at 72 hours post drug withdrawal following prior exposure to 2.5 μM GNF351 or 1 μM FICZ for 24, 48, or 72 hours. **F**-**G **Surface pan–HLA-II expression in WM115 (**F**) and SKMEL2 (**G**) cells at multiple timepoints post drug withdrawal, following treatment as in (**D**-**E**). **H**-**I **Surface pan–HLA-II expression in AHR or ARNT KO WM115 (**H**) and SKMEL2 (**I**) after 72-hour treatment with 1 μM FICZ or DMSO control. Surface pan–HLA-II expression was detected using APC anti-human HLA-DR, DP, DQ Antibody (clone Tü39). Representative histograms from three independent experiments show pan–HLA-II expression, with dashed lines indicating the median of the DMSO control group (**A**, **B** and **D**). The gMFI was quantified across three independent experiments, normalized to the DMSO control group (**C** and **E**-**G**) or to the mean of the two NTC sgRNAs with DMSO treatment (**H**-**I**), and presented as fold change. Data are represented as mean ± SD (**C** and **E**-**I**). Statistical analysis by one-way ANOVA (C and E); *p < 0.05, **p < 0.01, ***p < 0.001, ****p < 0.0001

To further investigate the functional role of the AHR-ARNT complex in regulating HLA-II expression, we treated human melanoma cells with an exogenous selective AHR antagonist GNF351, which inhibits AHR activity, and an endogenous tryptophan-derived potent AHR agonist FICZ, which activates AHR signaling. GNF351 treatment led to a marked decrease in HLA-II expression across all three human melanoma lines, including SKMEL2, which expresses relatively low basal levels of HLA-II (Fig. 3A). In the absence of IFN-γ stimulation, FICZ treatment significantly increased surface HLA-II expression in WM115 and SKMEL2 cells (Fig. 3A). In contrast, A375 cells, characterized by high basal HLA-II expression, exhibited minor upregulation upon FICZ treatment (Fig. 3A), possibly due to saturation of the regulatory pathway. In line with prior genetic experiments, treatment with FICZ failed to induce HLA-II expression in B16F10, MC38, and LLC (Supplementary Fig. 3B), underscoring the non-conservative nature of AHR-ARNT-mediated HLA-II regulation in humans and mice.

We next examined whether the magnitude of AHR-mediated regulation was dependent on the duration of ligand exposure. Time-course analysis revealed a progressive decrease or increase in surface HLA-II expression levels following treatment with GNF351 or FICZ, respectively, with greater changes observed after longer treatment (Fig. 3B-C). These results indicate that HLA-II expression on cancer cells could be modified through pharmacologic modulation of AHR activity, and that both the direction and magnitude of this regulation are time-dependent.

To evaluate the persistence of these regulatory effects, we measured surface HLA-II levels following withdrawal of GNF351 or FICZ. In A375 cells, GNF351 withdrawal was followed by a further decline in surface HLA-II levels after 72 h (Fig. 3D-E). Similar patterns were observed in WM115 and SKMEL2 cell lines (Fig. 3F-G). We observed that six days post-withdrawal, HLA-II expression levels showed the most pronounced downregulation or upregulation in the GNF351-treated and FICZ-treated groups, respectively (Fig. 3F-G). Thereafter, HLA-II levels gradually returned to baseline (Fig. 3F-G). These findings suggest that AHR modulation elicits lasting, but reversible, effects on HLA-II levels.

To confirm that FICZ-induced HLA-II upregulation is AHR-ARNT-dependent, we treated *AHR* and *ARNT* KO cells with FICZ. While slight increases in surface HLA-II were observed in *AHR* and *ARNT* KO cells, expression levels remained lower than those in FICZ-treated non-targeting controls (Fig. 3H-I), supporting the requirement for intact AHR-ARNT signaling. The residual FICZ-induced HLA-II increases observed in KO cells likely reflect incomplete knockout efficiency. Together, these results confirm that the AHR-ARNT complex mediates ligand-responsive, reversible regulation of HLA-II expression in human melanoma cells.

Based on the observed effects of AHR-ARNT on HLA-II, we next sought to determine whether similar regulatory patterns exist for HLA-I. Firstly, we examined basal and IFN-γ–induced HLA-I levels in A375, WM115, and SKMEL2 melanoma cells (Supplementary Fig. 4A). All three cell lines expressed high basal HLA-I, and IFN-γ treatment robustly increased HLA-I surface expression. We then examined the impact of AHR or ARNT knockout on HLA-I expression (Supplementary Fig. 4B). In A375 cells, knockout of AHR or ARNT did not significantly alter HLA-I expression. In contrast, AHR deletion in WM115 cells led to reduced HLA-I expression, whereas knockout of either gene in SKMEL2 cells resulted in a significant increase, indicating cell line–specific effects. Consistent with these findings, overexpression of AHR or ARNT had no appreciable impact on HLA-I levels in A375 cells but increased HLA-I expression in WM115 cells, while ARNT overexpression reduced HLA-I expression in SKMEL2 cells (Supplementary Fig. 4C). However, pharmacological inhibition of AHR with GNF351 consistently decreased HLA-I expression across all three cell lines, whereas activation of AHR with FICZ increased HLA-I expression in WM115 and SKMEL2 cells but not in A375 cells (Supplementary Fig. 4D). Collectively, these data indicate that, unlike its robust and consistent regulation of HLA-II, the impact of AHR-ARNT signaling on HLA-I expression is variable and context dependent, likely reflecting the dominant role of NLRC5 and other regulatory mechanisms governing HLA-I transcription in different cellular contexts.

### AHR-ARNT regulates HLA-II expression through transcriptional control of *CIITA*

**Fig. 4:**
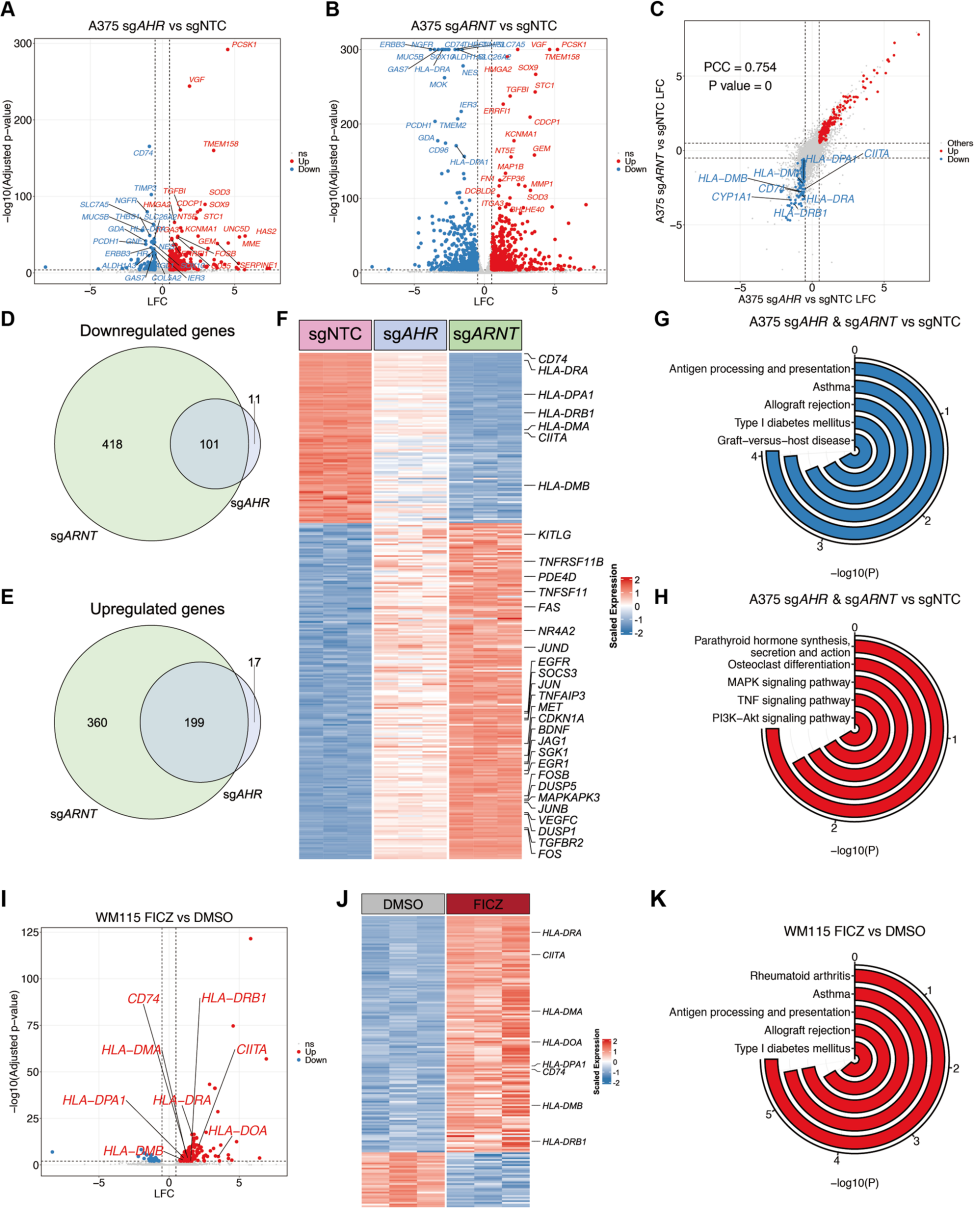
AHR signaling modulates HLA-II expression via transcriptional regulation of *CIITA. ***A**-**B **Scatterplots showing differentially expressed genes in A375 cells with CRSIPR-mediated knockouts of AHR (**A**) or ARNT (**B**) compared to NTC cells. Significantly downregulated (blue) and upregulated (red) genes are defined by adjusted *p *< 0.0001 and |log2 fold change| > 0.5. The top 20 differentially expressed genes are indicated. **C **Correlation analysis of gene expression changes between AHR KO and ARNT KO groups. Each point represents a gene; commonly upregulated (red) and downregulated (blue) genes are highlighted, with CYP1A1 and HLA-II-related genes indicated. **D**-**E **Venn diagrams showing the overlap of significantly downregulated (**D**) and upregulated genes (**E**) between AHR KO and ARNT KO groups. **F **Heatmap showing the common significantly downregulated and upregulated genes in AHR KO, ARNT KO, and NTC samples of A375, with HLA-II genes and cellular stress and signaling response genes indicated. **G**-**H **KEGG pathway enrichment analysis of common significantly downregulated genes (**G**) and upregulated genes (**H**) in AHR KO and ARNT KO groups. **I **Scatterplot showing differentially expressed genes in WM115 cells treated with 1 μM FICZ for 72 h compared to the 0.1% DMSO control group. Significantly downregulated (blue) and upregulated (red) genes are defined by adjusted *p* < 0.01 and |log2 fold change| > 0.5. HLA-II-related genes are indicated. **J **Heatmap of differentially expressed genes in WM115 cells with FICZ versus DMSO control, with HLA-II-related genes annotated. **K **KEGG analysis of significantly upregulated genes in the FICZ-treated group compared to the DMSO control group of WM115. Statistical analysis by t-test (C) and hypergeometric test (G-H and K). PCC, Pearson correlation coefficient

Given the ligand-responsiveness of HLA-II regulation by AHR-ARNT complex, we next sought to investigate the underlying molecular mechanisms. We performed transcriptomic profiling (RNA-seq) of A375 cells following CRISPR-mediated KO of either *AHR* or *ARNT*, and compared them to non-targeting controls. Differential gene expression analysis revealed broad transcriptional changes in both KO lines (Fig. 4A-B), with substantial overlap between the *AHR* and *ARNT* KO groups (Fig. 4C-E). This high concordance between *AHR* and *ARNT* KO profiles supports the cooperative function of this heterodimer in gene regulation. Notably, *CIITA* was consistently downregulated due to *AHR*- and *ARNT*-deficiency (Fig. 4C and F), suggesting the transcriptional control of *CIITA* by AHR-ARNT complex. Gene set enrichment analysis revealed that commonly downregulated genes were significantly enriched for pathways related to antigen processing and presentation (Fig. 4F-G). Gene sets related to cellular stress and signaling response, including components of the AP-1 complex (*FOS*, *FOSB*, *JUN*, *JUNB*, *JUND*), were significantly enriched among genes commonly upregulated in *AHR*- and *ARNT*-deficient cells compared to control cells (Fig. 4F and H).

To further determine whether AHR activation was sufficient to induce HLA-II-related gene expression, we treated WM115 cells with FICZ and performed RNA-Seq. Consistent with our findings from the KO models, FICZ treatment upregulated multiple HLA-II genes and *CIITA* (Fig. 4I-J). KEGG pathway analysis confirmed significant enrichment of the antigen processing and presentation pathway (Fig. 4J-K). RNA-seq analysis of WM115 cells following AHR or ARNT knockout (Supplementary Fig. 5A-F) and of A375 cells treated with FICZ (Supplementary Fig. 5G-H) yielded concordant results, but overexpression of AHR or ARNT did not significantly induce transcription of HLA-II genes in SKMEL2 cells (Supplementary Fig. 5I-K). Collectively, our findings support a model in which the AHR-ARNT complex regulates HLA-II expression by modulating *CIITA* transcription.

### The AHR-ARNT complex binds promoter II of *CIITA* to regulate its transcription

**Fig. 5:**
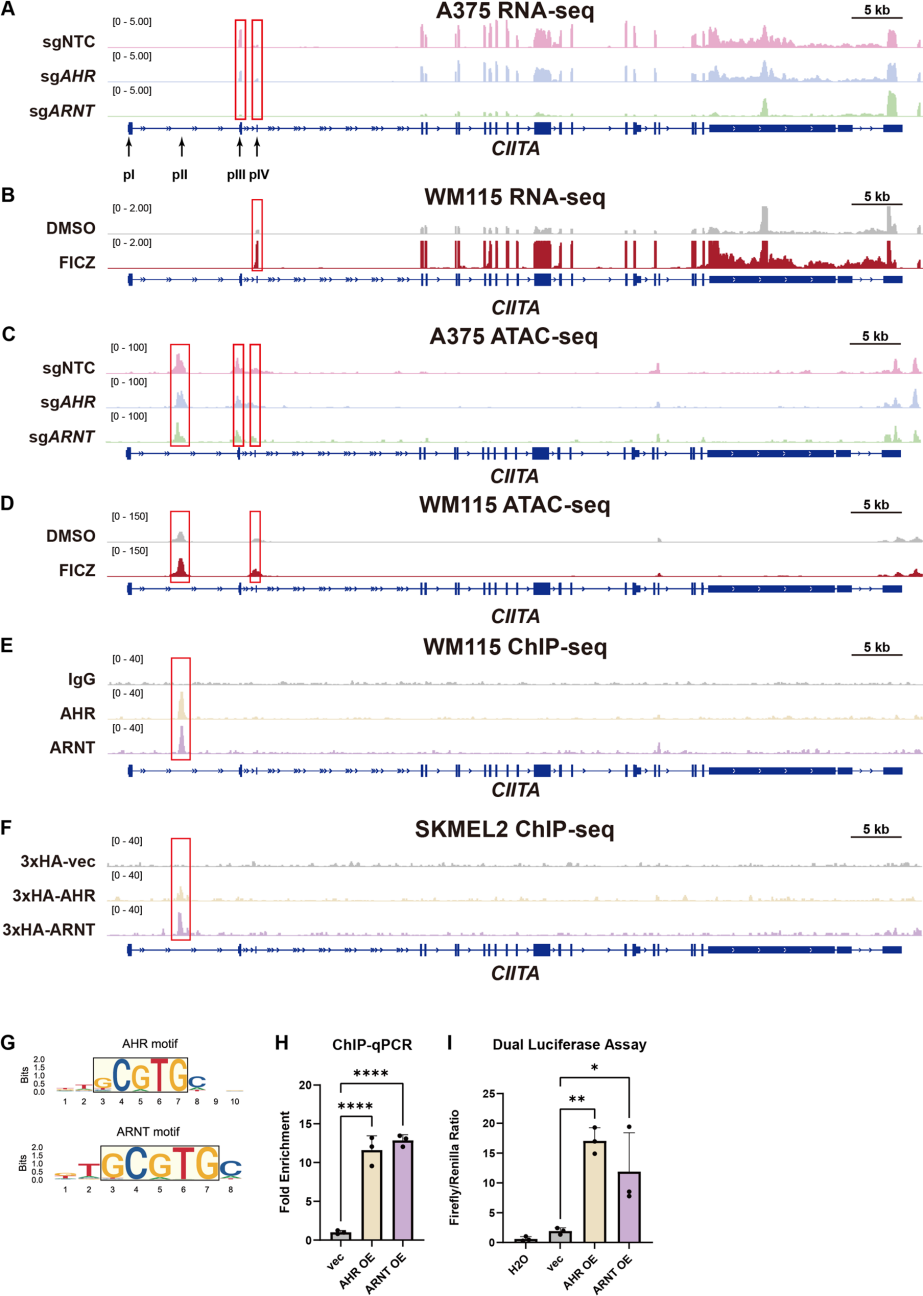
AHR-ARNT complex binds *CIITA* promoter II to drive HLA-II transcription. **A**-**B **Genome browser tracks showing gene CIITA mRNA expression (RNA-Seq) across the CIITA locus in A375 with sgNTC, sgAHR, or sgARNT (A) and in WM115 cells treated with 1 μM FICZ or 0.1% DMSO for 72 h (B). **C**-**D **ATAC-seq tracks showing chromatin accessibility at the CIITA locus in A375 cells with sgNTC, sgAHR, or sgARNT (**C**) and in WM115 cells treated with 1 μM FICZ or 0.1% DMSO for 72 h (**D**). **E**-**F **ChIP-seq tracks showing AHR or ARNT binding at the CIITA locus in SKMEL2 cells expressing empty vector control, 3×HA-AHR overexpression, or 3×HA-ARNT overexpression constructs, immunoprecipitated with anti-HA antibody (clone 1F5C6) (**E**) and in WM115 cells treated with 1 μM FICZ for 72 h, immunoprecipitated with IgG control (clone DA1E), anti-AHR (clone D5S6H), or anti-ARNT (clone D28F3) antibodies (**F**). **G **Enrichment analysis of transcription factor motifs in AHR- and ARNT-binding regions identified by ChIP-seq of SKMEL2 cells overexpressing 3×HA-tagged AHR or ARNT. Top-enriched motifs include canonical AHR response elements. **H **ChIP-qPCR quantification of AHR or ARNT occupancy at the CIITA promoter II (pII) region in SKMEL2 cells expressing empty vector control, 3×HA-AHR overexpression, or 3×HA-ARNT overexpression constructs. Enrichment is shown relative to input and normalized to the empty vector control. **I **Luciferase reporter assay using a construct containing the CIITA pII co-transfected with Renilla luciferase control into 293T cells expressing empty vector control, AHR overexpression, or ARNT overexpression constructs. vec, empty vector control. Data are represented as mean ± SD (**H**-**I**). Each dot represents one technical replicate from triplicate wells (**H**-**I**). Statistical analysis by one-way ANOVA (**H**-**I**); *p < 0.05, **p < 0.01, ***p < 0.001, ****p < 0.0001. vec, empty vector control

To elucidate how the AHR-ARNT complex regulates HLA-II expression at the transcriptional level, we analyzed *CIITA* mRNA isoforms using RNA-seq. In A375 cells, *CIITA* transcripts originated predominantly from the type III 5’ end and, to a lesser extent, from the type IV 5’ end (Fig. 5A), consistent with prior reports describing abnormal usage of the B cell-specific promoter pIII in A375 melanoma cells [16]. In contrast, *CIITA* transcription initiated from the type IV 5’ end in WM115 cells (Fig. 5B), indicating that *CIITA* promoter usage varies by cell type. Moreover, ablation of *AHR* or *ARNT* significantly reduced type III and IV transcript levels in A375 cells (Fig. 5A), while FICZ treatment increased type IV transcript abundance in WM115 cells, suggesting a potential involvement of AHR-ARNT in the regulation of pIII and pIV.

In order to further investigate the regulatory mechanism, ATAC-seq was performed. Consistent with the RNA-seq data, ATAC-seq showed that chromatin accessibility at the HLA-DRA promoter was reduced in *AHR* and *ARNT* KO cells, and was induced after FICZ treatment (Supplementary Fig. 6A-B). Notably, promoters pII, pIII, and pIV were accessible, and their accessibility remained largely unchanged regardless of *AHR/ARNT* status or pharmacological treatment in A375 and SKMEL2 cells (Fig. 5C and Supplementary Fig. 6C-D). In WM115, pII and pIV were accessible under all conditions, showing a slight increase after FICZ treatment and a mild reduction following AHR or ARNT knockout (Fig. 5D and Supplementary Fig. 6E). However, pIII was not accessible in WM115 cells under any condition (Fig. 5D and Supplementary Fig. 6E). These findings suggest that AHR-ARNT does not primarily regulate CIITA through global chromatin remodeling but instead acts at the level of promoter-specific transcriptional engagement.

To directly test this possibility, we performed ChIP-seq in A375 and WM115 cells using antibodies against endogenous AHR and ARNT, and in SKMEL2 cells overexpressing 3×HA-tagged AHR or ARNT using an HA antibody. As expected, the canonical AHR-ARNT target gene *CYP1A1* exhibited strong promoter enrichment (Supplementary Fig. 6F-H). Importantly, AHR-ARNT binding was consistently detected at the *CIITA* pII region across all cell lines, whereas no enrichment was observed at pIII or pIV (Fig. 5E-F and Supplementary Fig. 6I). These findings identify pII as a direct and conserved AHR-ARNT target element in melanoma cells, and suggest that, rather than initiating pII-driven transcription, AHR-ARNT primarily enhances transcription from other *CIITA* promoters.

Motif enrichment analysis using MEME-ChIP [35] revealed that the AHRE (Aryl Hydrocarbon Response Element) core motif (5’-GCGTG-3’) was the most significantly enriched motif in the ChIP peaks for both factors (Fig. 5G). Notably, this motif is present in the pII region of *CIITA*. We further validated AHR-ARNT binding to pII using ChIP-qPCR in HA-AHR/ARNT-overexpressing SKMEL2 cells (Fig. 5H), confirming direct occupancy at this site. To assess the functional activity of this binding site, we PCR cloned the enriched pII region into a dual-luciferase reporter construct. Co-transfection with AHR and ARNT significantly increased reporter activity, confirming its role as a transcriptionally active AHR-ARNT-responsive element (Fig. 5I). Together, these results demonstrate that AHR directly regulates CIITA transcription through pII, providing a mechanistic explanation for the species-specific effects observed earlier, as mice lack the CIITA pII promoter [14].

Finally, RT-qPCR using isoform-specific primers revealed that AHR inhibition by GNF351 suppressed expression of type III *CIITA* mRNA isoforms across all cell lines (Supplementary Fig. 7A-C), and suppressed type IV expression in WM115 cells (Supplementary Fig. 7B). AHR activation by FICZ induced type III and IV isoforms in WM115 and SKMEL2 cells, whereas induction of type II isoform was only observed in SKMEL2 cells (Supplementary Fig. 7B-C). Collectively, these results indicate that although AHR-ARNT directly binds the CIITA pII promoter, AHR signaling broadly influences CIITA isoform expression, including pIII- and pIV-driven transcripts, likely through indirect regulatory mechanisms.

### *AHR-ARNT*-KO signature negatively correlates with clinical benefits in multiple cancer cohorts

To evaluate the clinical relevance of the AHR-ARNT regulatory axis in human tumors, we developed a gene expression signature reflective of AHR-ARNT functional loss by identifying genes consistently dysregulated upon loss of either AHR or ARNT. A composite score was then calculated for each tumor sample using a weighted sum–based approach, in which gene expression values were multiplied by normalized weights derived from DESeq2 Wald statistics (Supplementary File 1), enabling estimation of AHR-ARNT pathway activity in large clinical datasets.

**Fig. 6:**
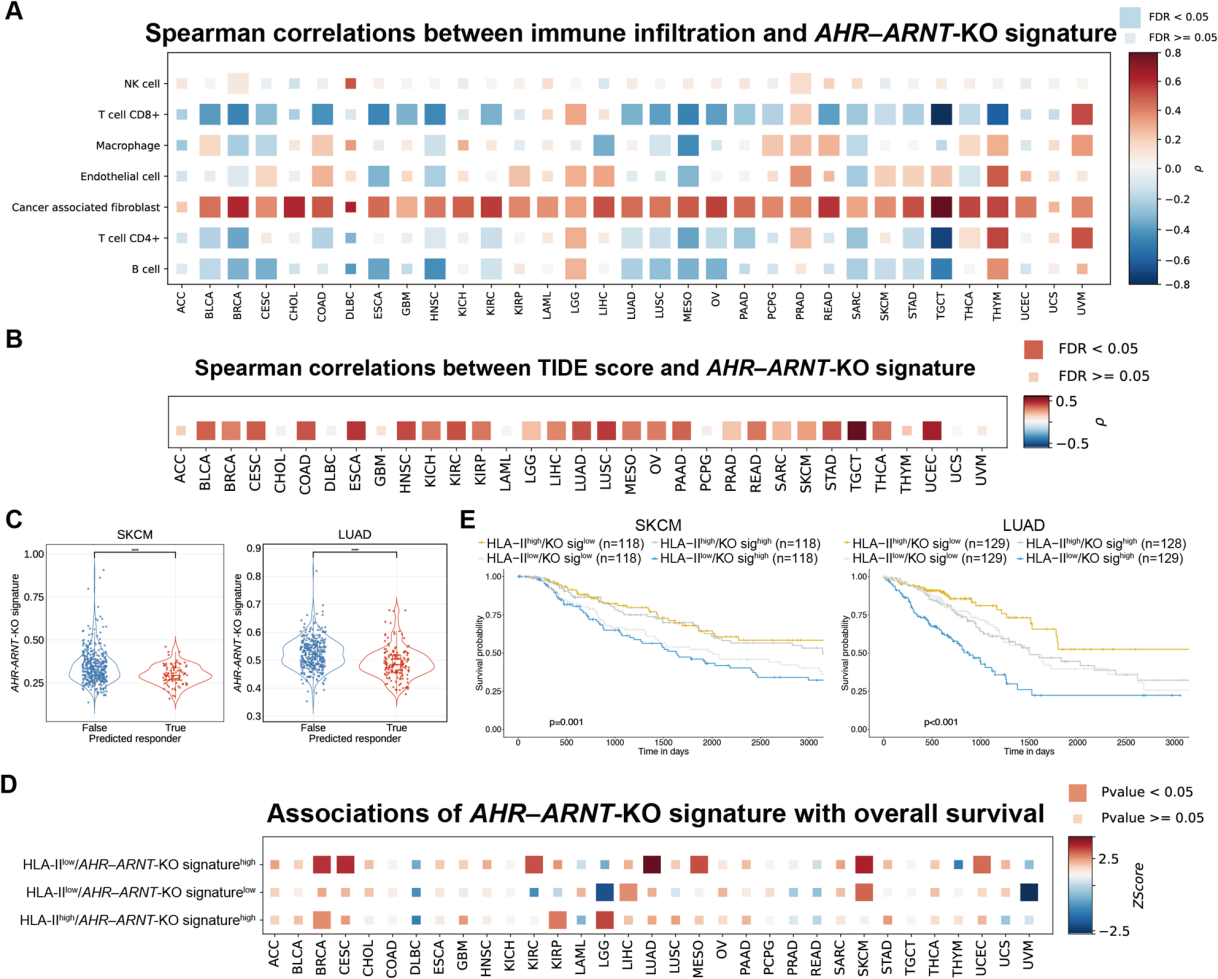
Clinical relevance of AHR pathway activity with tumor immunity and patient outcome. **A **Heatmap showing Spearman correlation coefficients between infiltrating immune cell levels and AHR‒ARNT-KO signature scores across cancer types in The Cancer Genome Atlas (TCGA) cohorts. **B **Heatmap of Spearman correlations between Tumor Immune Dysfunction and Exclusion (TIDE) scores and AHR‒ARNT-KO signature scores across TCGA cohorts. **C **Distribution of AHR‒ARNT-KO signature scores predicted by TIDE in non-responders and responders from the TCGA skin cutaneous melanoma (SKCM) and lung adenocarcinoma (LUAD) cohorts. SKCM: non-responders, n=381; responders, n=92. LUAD: non-responders, n=378; responders, n=198. **D **Heatmap showing associations between overall survival and combined HLA-II expression and AHR‒ARNT-KO signature groups across TCGA cohorts. Multivariable Cox proportional hazards model adjusted for age, gender, and tumor stage were used to compute z-scores and p-values; the HLA-IIhigh/AHR‒ARNT-KO signaturelow group is used as reference. **E **Kaplan-Meier survival analysis of SKCM and LUAD patients from TCGA, stratified by HLA-II expression and AHR‒ARNT-KO signature scores. The p-values shown correspond to the comparison between the HLA-IIlow/AHR‒ARNT-KO signaturehigh and HLA-IIhigh/AHR‒ARNT-KO signaturelow groups and were adjusted using the Benjamini–Hochberg method. Statistical analysis by unpaired Student’s t-test (**C**) and log-rank test (**E**); *p < 0.05, **p < 0.01, ***p < 0.001, ****p < 0.0001

We first assessed the association between the *AHR-ARNT*-KO signature and immune cell infiltration using bulk RNA-seq data from The Cancer Genome Atlas (TCGA). Across multiple TCGA cohorts, higher *AHR-ARNT*-KO signature scores were significantly correlated with reduced infiltration of CD4⁺ T and CD8⁺ T cells and B cells, and with increased infiltration of cancer-associated fibroblasts, as estimated by EPIC [36] (Fig. 6A). To explore potential implications for immunotherapy responsiveness, we applied the TIDE algorithm [37] to the TCGA datasets. In 24 cancer types, including skin cutaneous melanoma (SKCM) and lung adenocarcinoma (LUAD), patients predicted to respond to immune checkpoint blockade (ICB) exhibited significantly lower *AHR-ARNT*-KO signature scores than predicted non-responders (Fig. 6B-C). These findings suggest that intact AHR-ARNT activity may contribute to an immunologically active tumor microenvironment that is more amenable to ICB therapy.

We next examined the association between the *AHR-ARNT*-KO signature, HLA-II expression, and patient survival. For each TCGA cancer type, patient samples were stratified into four groups based on high or low HLA-II expression level and high or low *AHR-ARNT*-KO signature scores. Multivariable Cox proportional hazards analyses adjusted for age, gender, and tumor stage revealed that, in a subset of cancer types including SKCM and LUAD, patients with high HLA-II expression and low *AHR-ARNT*-KO signature scores exhibited a significantly lower risk of mortality (Fig. 6D). Consistent with these findings, Kaplan–Meier analyses in both SKCM and LUAD cohorts demonstrated significantly longer overall survival for this group compared to patients with low HLA-II expression and high *AHR-ARNT*-KO signature scores (Fig. 6E). These associations underscore the relevance of AHR-ARNT-mediated regulation of HLA-II to the tumor immune landscape and its association with patient outcomes.

## Discussion

The regulation of HLA-II expression in cancer cells is increasingly recognized as a critical determinant of TME and responsiveness to immunotherapy [3–6, 18–21]. Although antigen presentation by HLA-II is canonically restricted to pAPCs, accumulating evidence has demonstrated that HLA-II expression on cancer cells correlates with improved patient prognosis and enhanced response to ICB. Recent studies have identified several negative regulators of HLA-II expression, including FBXO11 and PRMT1, which promote degradation of CIITA through ubiquitination and methylation, respectively, as well as the CtBP complex, which represses HLA-II transcription [26,27]. However, these regulators act downstream of CIITA, the master transcriptional activator of HLA-II, and require its presence to exert their effects. In contrast, the upstream mechanisms that drive constitutive HLA-II and CIITA in non-hematopoietic tumors such as melanoma remain incompletely defined. In this study, we employed genome-wide CRISPR screening in human melanoma cell lines (A375 and WM115) and uncovered a previously unrecognized regulatory axis involving the AHR and its obligate dimerization partner ARNT. Together, the AHR-ARNT complex functions as a key positive regulator of HLA-II expression on cancer cells. Notably, this regulation is observed in the absence of exogenous IFN-γ stimulation, indicating that the AHR-ARNT complex contributes to the control of basal HLA-II expression on tumor cells and may complement inflammatory cytokine–driven pathways in regulating tumor cell–associated antigen presentation.

AHR has been classically characterized as a mediator of cellular responses to environmental toxins, including dioxins and polycyclic aromatic hydrocarbons, primarily through induction of detoxifying enzymes such as cytochrome P450 family members [29]. Beyond xenobiotic metabolism, AHR has emerged as a multifaceted regulator of immune function, influencing T cell differentiation, epithelial homeostasis, and cellular proliferation and development [30–32]. In this study, we extend the functional repertoire of AHR by identifying the AHR-ARNT complex as a previously unrecognized regulator of HLA-II expression on tumor cells. As a ligand-activated transcription factor that senses and responds to diverse endogenous and exogenous ligands, AHR is well positioned to couple tissue perturbation to immune surveillance. Accordingly, AHR-driven induction of HLA-II expression may represent an adaptive mechanism by which transformed cells increase their visibility to the immune system. In this context, AHR signaling could confer an immune advantage in tumor microenvironments.

The significant reduction in HLA-II expression following KO of *AHR* or *ARNT* underscores the essential role of this complex in sustaining CIITA-dependent HLA-II expression on cancer cells. Conversely, overexpression of AHR or ARNT was sufficient to induce HLA-II upregulation even in the absence of exogenous IFN-γ. While HLA-II traditionally presents exogenous peptides processed by pAPCs, several alternative pathways enable endogenous antigen loading onto HLA-II molecules [38–42]. These mechanisms suggest that cancer cells have a route to present tumor-associated antigens (TAAs) or tumor-specific antigens (TSAs) on their own HLA-II molecules, thereby contributing to direct CD4 + T cell activation [43–50]. Thus, the AHR-ARNT-dependent regulation of basal HLA-II provides a mechanistic framework through which tumor cells may acquire antigen-presenting capacity.

Our results reveal that the regulation of the AHR and ARNT pathway is both ligand-responsive and temporally dynamic. Treatment with FICZ, a potent AHR agonist, significantly increased surface HLA-II expression across multiple melanoma lines, while AHR inhibition with GNF351 led to its repression. Importantly, HLA-II upregulation persisted and peaked after FICZ withdrawal, suggesting a sustained transcriptional effect downstream of transient AHR activation. This temporal feature has important implications for the design and scheduling of AHR-targeted interventions, potentially facilitating their therapeutic exploitation in cancer immunotherapy.

Mechanistically, we demonstrate that the AHR-ARNT complex directly binds to promoter II (pII) of *CIITA*, thereby enhancing its transcriptional activity. This establishes a direct link between environmental sensing via AHR ligands and the activation of antigen presentation machinery in tumor cells. The presence of a canonical AHRE within the *CIITA* pII, together with robust ChIP-seq/ChIP-qPCR validation, reinforces the specificity of this regulation. In A375 cells, AHR or ARNT deficiency led to reduced abundance of type III and IV mRNA of *CIITA*, suggesting that AHR-ARNT may influence *CIITA* transcription through promoter-binding–independent regulation, potentially involving promoter switching or epigenetic modulation. As we continue to delineate the molecular pathways governing HLA-II regulation, it will be critical to explore potential combinatorial strategies that integrate AHR pathway modulation with approaches aimed at enhancing T cell activation and persistence. Insights from this study provide a mechanistic basis for exploring the AHR-ARNT axis in the context of cancer immunotherapy.

Our findings also underscore the complex, context-dependent role of AHR in cancer. While AHR activation has previously been implicated in both tumor-promoting and tumor-suppressive processes [30, 51, 52], our study reveals a novel immune-enhancing function through upregulation of HLA-II expression. By contrast, the impact of AHR-ARNT signaling on HLA-I expression is variable and cell line–dependent, highlighting important contextual constraints on its broader immunoregulatory effects. This duality underscores the necessity for a nuanced comprehension of AHR signaling across various tumor types, disease stages, and immune microenvironments. Notably, should AHR prove capable of coordinately upregulating both HLA-I and HLA-II in specific settings, the resulting enhancement of antigen presentation could synergize with immune checkpoint blockade. Future studies should therefore carefully delineate the conditions under which AHR modulation promotes versus impairs anti-tumor immunity, with particular attention to its interactions with other immunoregulatory pathways.

In addition, the discrepancy between the effects of AHR-ARNT overexpression or activation on human and mouse cancer cells may reflect fundamental differences in the structure and downstream signaling of the AHR-ARNT pathway between humans and mice [51], including species-specific variation in ligand affinity, transcriptional targets, and chromatin context [53–58]. In addition, the pII promoter of CIITA is not conserved in mice [14], providing a potential mechanistic explanation for the lack of functional conservation. Together, these findings establish AHR-ARNT as a selective and critical regulator of HLA-II expression in human melanoma cells, with species-specific constraints that limit its activity in murine models.

In clinical datasets, higher inferred AHR-ARNT activity, based on an AHR-ARNT loss-of-function signature, was associated with increased immune cell infiltration, better response to ICB, and improved overall survival across multiple cancer types. These correlative findings suggest that the AHR-ARNT pathway may serve not only as a functional modulator but also as a potential biomarker associated with favorable immunotherapy outcomes. Notably, however, the retrospective nature of these analyses precludes definitive conclusions regarding predictive value. Prospective or longitudinal cohorts with well-defined therapeutic exposure will be required to determine whether activation of the AHR–CIITA–HLA-II axis directly predicts response to specific treatments, including ICB or targeted therapies. Consistent with the established role of HLA-II in promoting durable antitumor immunity, pharmacological enhancement of HLA-II expression on cancer cells via AHR agonists may augment T cell-mediated responses. Further studies are warranted to explore the combinatorial potential of AHR activation with immune checkpoint inhibitors and to dissect the interplay between AHR-ARNT and other immunomodulatory pathways.

**Fig. 7:**
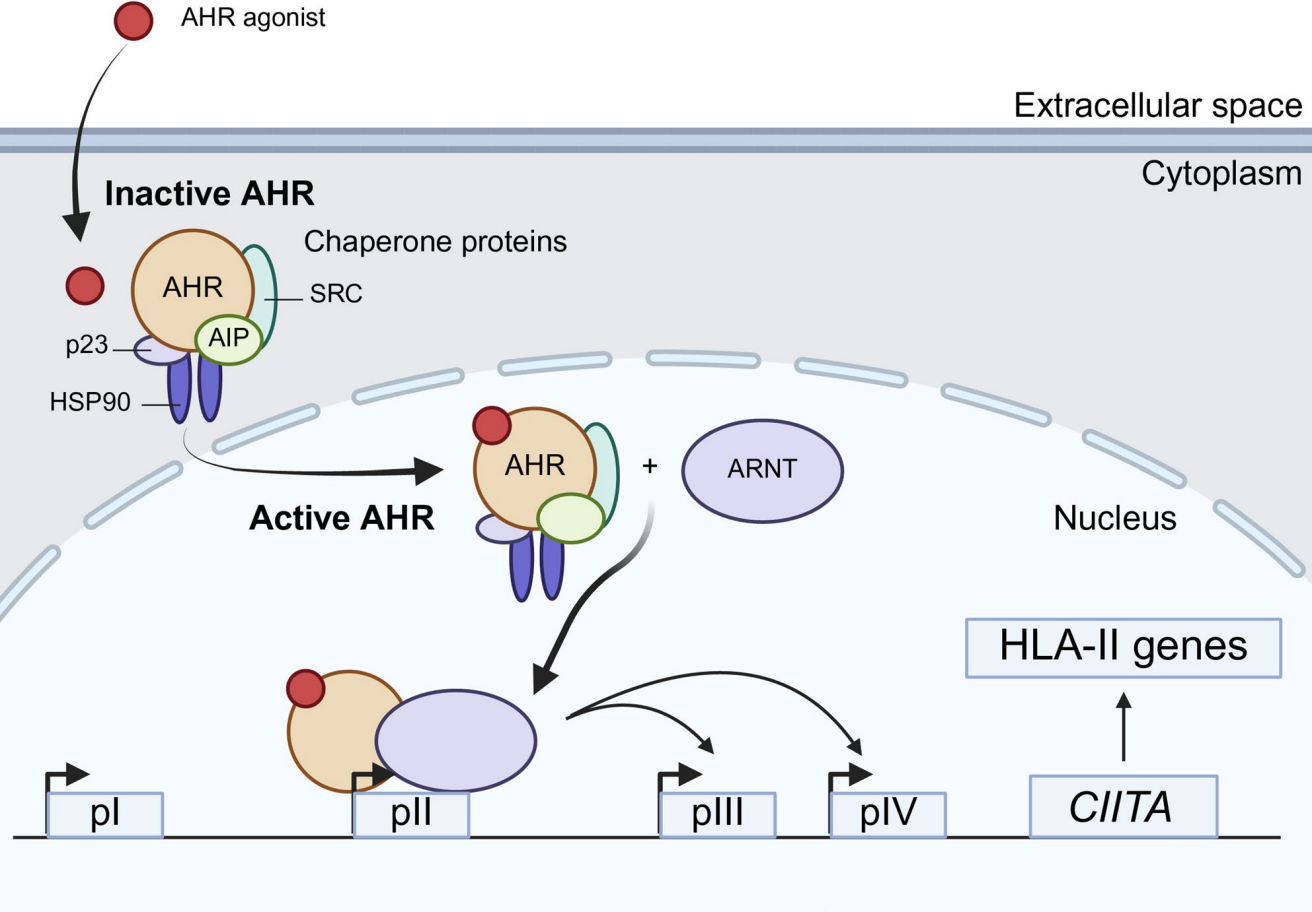
Schematic model illustrating transcriptional activation of CIITA via pII by ligand-activated AHR-ARNT complex

Based on our experimental findings and established knowledge of AHR biology, we propose a model in which ligand activation of AHR promotes formation of an AHR-ARNT transcriptional complex that engages the CIITA pII promoter to regulate CIITA transcription and downstream HLA-II expression (Fig. 7). In this model, direct association of AHR with the CIITA pII promoter is supported by our ChIP and transcriptional analyses, whereas nuclear translocation of AHR and its heterodimerization with ARNT are inferred from well-established mechanisms described in prior studies [29]. Together, this model provides a framework linking ligand-responsive AHR signaling to CIITA-dependent regulation of antigen presentation in tumor cells.

Despite the insights provided by this study, several limitations should be considered. First, although our data support direct association of the AHR-ARNT complex with the CIITA pII promoter, the functional requirement of individual AHR-binding motifs has not been formally validated by site-directed mutagenesis. Second, the observed species-specific differences between human and murine tumor cells highlight an important limitation of current preclinical models and emphasize the need for human-relevant systems to study tumor-associated antigen presentation. Finally, while pharmacological modulation of AHR activity yielded clear mechanistic insights, the pleiotropic effects and potential toxicities of AHR-targeting compounds may complicate clinical translation, necessitating careful optimization of therapeutic strategies.

## Conclusions

In summary, our study identifies the AHR-ARNT complex as a central, ligand-responsive regulator of *CIITA* and HLA-II expression in human melanoma cells. By promoting transcriptional activation of *CIITA* via direct binding to its promoter II, AHR signaling enables tumor cells to acquire an antigen-presenting capacity, thereby increasing their immunogenicity and potential visibility to the immune system. The reversible and tunable nature of this regulatory mechanism presents opportunities for therapeutic intervention, particularly in combination with existing immunotherapies. More broadly, these findings underscore the significance of transcriptional programs within tumor cells that govern antigen presentation in shaping anti-tumor immunity and provide a foundation for future AHR-targeted strategies in cancer immunotherapy.

## Supplementary Information

Additional file 1.csv: sgRNA sequences used in this study.

Additional file 2.csv: ChIP-qPCR primer sequences used in this study.

Additional file 3.csv: RT-qPCR primer sequences used in this study.

Additional file 4.csv: AHR-ARNT-KO signature genes with weights, descriptions and annotation sources.

Additional file 5: Supplementary figures.

## Data Availability

The datasets supporting the conclusions of this article are available in the NCBI Gene Expression Omnibus (GEO) repository, GSE305296 (https://www.ncbi.nlm.nih.gov/geo/query/acc.cgi?acc=GSE305296). Transcriptome data and clinical data for TCGA cohorts were obtained from the TCGA Data Portal ( [https://portal.gdc.cancer.gov/](https:/portal.gdc.cancer.gov) ). No custom programs were developed specifically for this manuscript. Cell lines generated in this study will be provided by the lead contact under a material transfer agreement.

## Abbreviations

AHR: Aryl hydrocarbon receptor
AHREs: AHR response elements
ANOVA: Analysis of variance
ARNT: Aryl hydrocarbon receptor nuclear translocator
ATAC-seq: Assay for transposase-accessible chromatin using sequencing
BCA: Bicinchoninic acid
Cas9: CRISPR-associated protein 9
CCLE: Cancer Cell Line Encyclopedia
cDNA: Complementary DNA
CDS: Coding sequence
ChIP: Chromatin immunoprecipitation
ChIP-qPCR: ChIP followed by quantitative PCR
ChIP-seq: ChIP sequencing
CIITA: HLA class II transactivator
CRISPR: Clustered regularly interspaced short palindromic repeats
DAPI: 4′,6-diamidino-2-phenylindole
DEG: Differential gene expression
DMEM: Dulbecco’s Modified Eagle Medium
DMSO: Dimethyl sulfoxide
EPIC: Estimating the proportion of immune and cancer cells
FACS: Fluorescence-associated cell sorting
FASTQ: Format for storing nucleotide sequences and quality scores
FBS: Fetal bovine serum
FICZ: 6-formylindolo[3,2-b]carbazole
gMFI: Geometric mean fluorescence intensity
GO_BP: Gene Ontology Biological Process
gRNA: Guide RNA
HLA: Human leukocyte antigen
ICB: Immune checkpoint blockade
IFN-γ: Interferon gamma
KEGG: Kyoto Encyclopedia of Genes and Genomes
KO: Knockout
LUAD: Lung adenocarcinoma
mAb: Monoclonal antibody
MaGeCK: Model-based Analysis of Genome-wide CRISPR-Cas9 Knockout
MHC: Major histocompatibility complex
mRNA: Messenger RNA
NSCLC: Non-small cell lung cancer
NTC: Non-targeting control
OE: Overexpression
PAM: Protospacer adjacent motif
pan–HLA-I: HLA-A, B, C
pan–HLA-II: HLA-DR, DP, DQ
pAPCs: Professional antigen-presenting cells
PBS: Phosphate-buffered saline
PCC: Pearson correlation coefficient
PEI: Polyethylenimine
pI, pII, pIII, pIV: Promoter I, II, III, IV of CIITA
PVDF: Polyvinylidene fluoride
RFP: Red fluorescent protein
RNA-seq: RNA sequencing
RPGC: Reads per genomic content
RT-qPCR: Reverse transcription quantitative PCR
SD: Standard deviation
SDS-PAGE: Sodium dodecyl sulfate–polyacrylamide gel electrophoresis
sgRNA: Single guide RNA
SKCM: Skin cutaneous melanoma
STAR: Spliced transcripts alignment to a reference
TAAs: Tumor-associated antigens
TBST: Tris-buffered saline with Tween 20
TCGA: The Cancer Genome Atlas
TIDE: Tumor immune dysfunction and exclusion
TME: Tumor microenvironment
TNBC: Triple-negative breast cancer
TSAs: Tumor-specific antigens
vec: Empty vector control
