# Supplementary material for: The aryl hydrocarbon receptor (AHR) drives human leukocyte antigen (HLA)-II expression in human melanoma
Source: J Exp Clin Cancer Res. 2026 Feb 20;45:78. doi: 10.1186/s13046-026-03673-y (PMC13032475; doi:10.1186/s13046-026-03673-y)
Supplement: Supplementary file 5 — Additional file 5: Supplementary figures. [file 13046_2026_3673_MOESM5_ESM.pdf]

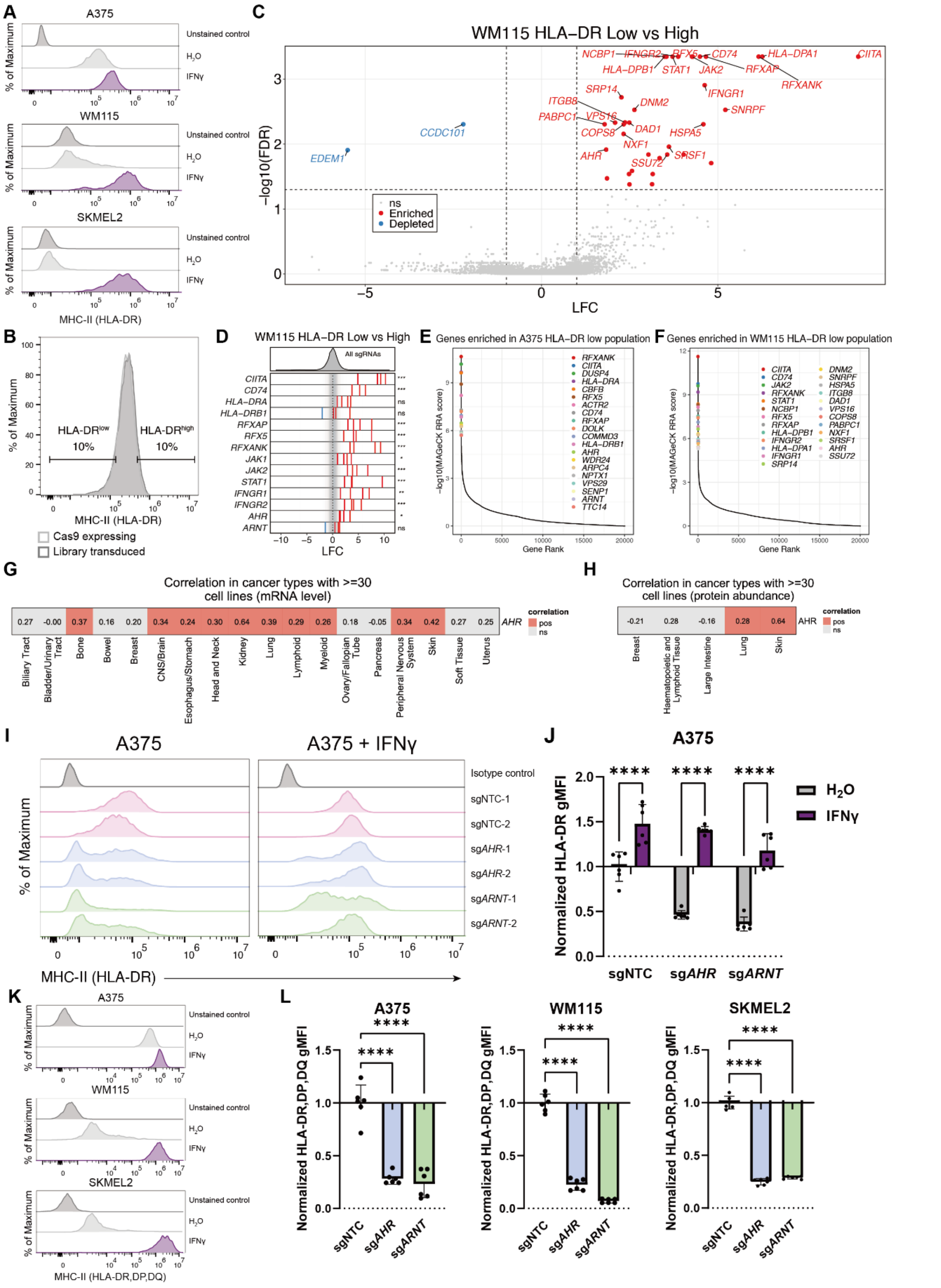

Supplementary Figure 1. Genome-wide CRISPR screens identify *AHR* and *ARNT* as positive regulators of HLA-II expression.

(**A**) Surface expression of HLA-DR in A375, WM115, and SKMEL2 cells treated with or without 100 ng/mL IFN-γ for 72 hours.

(**B**) Representative flow cytometry histograms from three independent experiments showing HLA-DR expression in A375 cells expressing Cas9 and transduced with the Brunello genome-wide gRNA library. Gates indicate the top 10% (HLA-DR^{high}) and bottom 10% (HLA-DR^{low}) populations sorted for downstream analysis.

(**C**) Scatterplot showing the top 25 enriched (red) and depleted (blue) sgRNAs in the HLA-DR^{low} population compared to the HLA-DR^{high} population in WM115.

(**D**) Log_2 fold change values of individual sgRNAs plotted in (**C**), showing the effect of gene perturbations on HLA-DR expression.

(**E-F**) Rank plots showing top 20 genes enriched in A375 HLA-DR^{low} population (**E**) and top 25 genes enriched in WM115 HLA-DR^{low} population (**F**).

(**G-H**) Heatmaps showing Pearson correlation coefficients between AHR and HLA-II expression at the mRNA level (**G**) and protein level (**H**) across cancer types with more than 30 cell lines in the CCLE.

(**I-J**) Surface expression of HLA-DR in A375-Cas9 cells transduced with sgRNAs targeting *AHR*, *ARNT*, or control sgRNAs, with or without IFN-γ treatment (100 ng/mL, 72 h).

(**K**) Surface expression of pan–HLA-II in A375, WM115, and SKMEL2 cells treated with or without 100 ng/mL IFN-γ for 72 hours.

(**L**) Surface expression of pan–HLA-II in *AHR*- and *ARNT*-deficient A375, WM115, and SKMEL2 cells.

Surface HLA-DR expression was detected using FITC anti-human HLA-DR Antibody (clone LN3) with FITC Mouse IgG2b, κ Isotype Ctrl Antibody (clone 27-35) as isotype control (**A, B, I, and J**). Surface pan–HLA-II expression was detected using APC anti-human HLA-DR, DP, DQ Antibody (clone Tü39) (**K and L**). Representative flow cytometry histograms from three independent experiments show HLA-DR (**A and I**) or pan–HLA-II (**K**) expression. The gMFI was quantified across three independent experiments, normalized to the NTC group without IFN-γ treatment and presented as fold change (**J and L**). Data from the two sgRNAs within each group were pooled and represented as mean ± SD (**J and L**). Statistical analysis by two-way ANOVA (**J**) and one-way ANOVA (**L**); ****$p < 0.0001$.

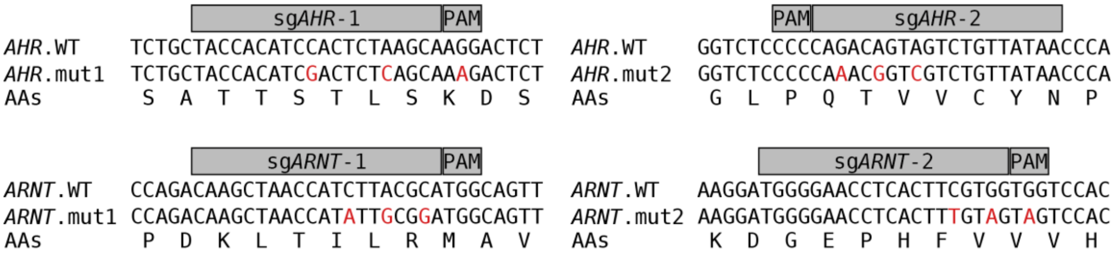

Supplementary Figure 2. Schematic of synonymous mutations in AHR/ARNT sgRNA target sites.

Schematic showing the introduction of three nonconsecutive synonymous nucleotide substitutions into the sgRNA target site and adjacent PAM sequence of *AHR* and *ARNT*, used to enable re-expression in knockout backgrounds without re-targeting.

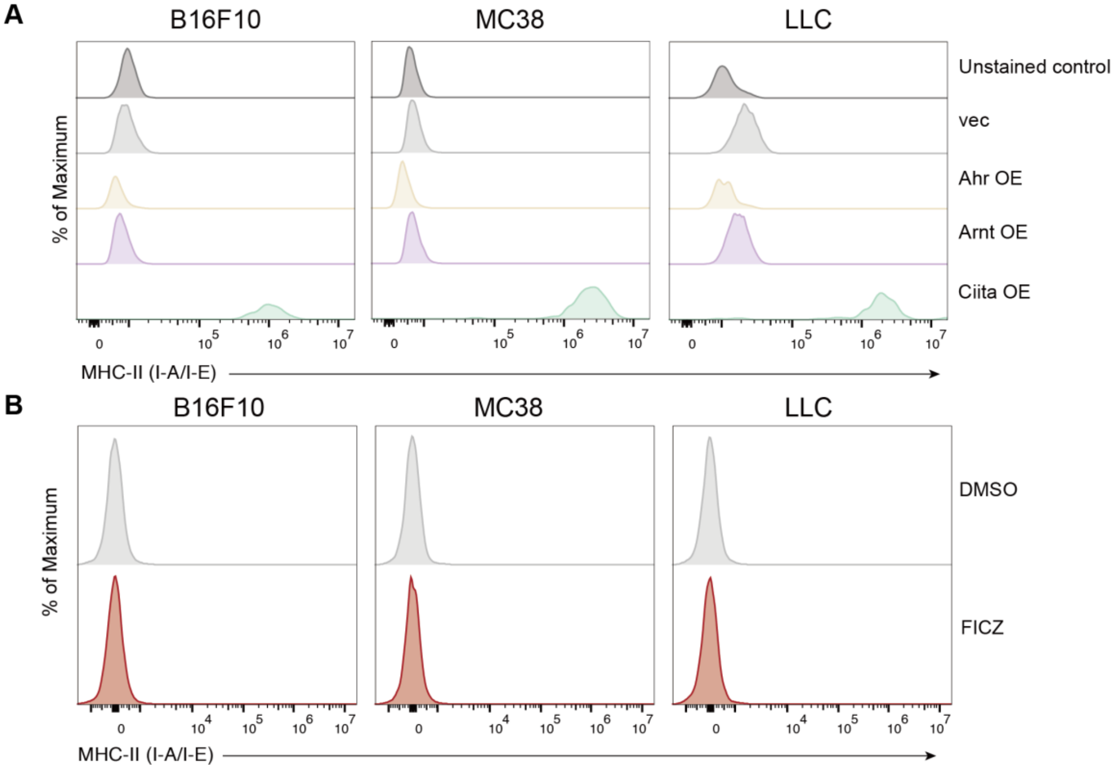

Supplementary Figure 3. Ahr/Arnt overexpression and activation fail to induce MHC-II expression in mouse cancer cells.

(**A**) Surface MHC-II (I-A/I-E) expression in murine tumor cell lines (B16F10, LLC, and MC38) following overexpression of Ahr, Arnt, or Ciita. Empty vector (vec) used as control.

(**B**) Surface MHC-II (I-A/I-E) expression in murine tumor cell lines (B16F10, LLC, and MC38) treated with 1 μM FICZ or 0.1% DMSO (vehicle control) for 72 hours.

Surface I-A/I-E expression was detected using FITC anti-mouse I-A/I-E Antibody (clone M5/114.15.2). Representative plots from three independent experiments. vec, empty vector control.

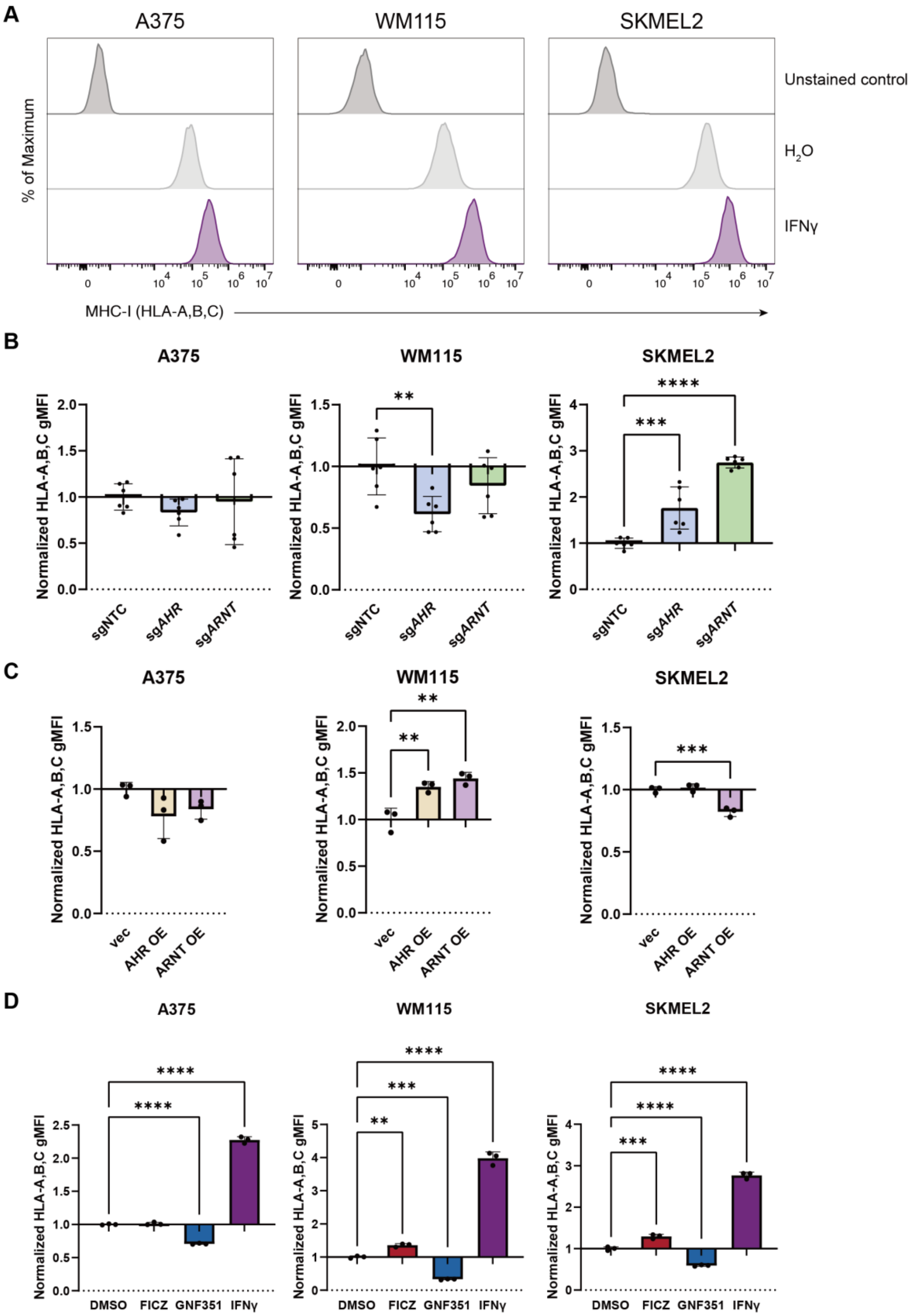

Supplementary Figure 4. The effect of AHR-ARNT signaling on HLA-I are variable and context-dependent.

(**A**) Surface expression of pan–HLA-I (HLA-A, B, C) in A375, WM115, and SKMEL2 cells treated with or without 100 ng/mL IFN-γ for 72 hours. Representative flow cytometry histograms from three independent experiments show pan–HLA-I expression.

(**B**) Surface expression of pan–HLA-I in *AHR*- and *ARNT*-deficient A375, WM115, and SKMEL2 cells. The gMFI was quantified across three independent experiments, normalized to the NTC group, and presented as fold change. Data from the two sgRNAs within each group were pooled.

(**C**) Surface expression of pan–HLA-I in A375, WM115, and SKMEL2 cells overexpressing AHR or ARNT. The gMFI was quantified across three independent experiments, normalized to the empty vector control group and presented as fold change.

(**D**) Surface expression of pan–HLA-I in A375, WM115, and SKMEL2 melanoma cells following 72-hour treatment with 2.5 μM GNF351 (AHR antagonist), 1 μM FICZ (AHR agonist), 100 ng/mL IFN-γ, or 0.1% DMSO (vehicle control). The gMFI was quantified across three independent experiments, normalized to the DMSO control group, and presented as fold change.

Surface pan–HLA-II expression was detected using PE anti-human HLA-A, B, C Antibody (clone W6/32) (**A-C**) and APC anti-human HLA-A, B, C Antibody (clone W6/32) (**D**). Data are represented as mean ± SD (**B-D**). Statistical analysis by one-way ANOVA (**B-D**); *p < 0.05, **p < 0.01, ***p < 0.001, ****p < 0.0001. vec, empty vector control.

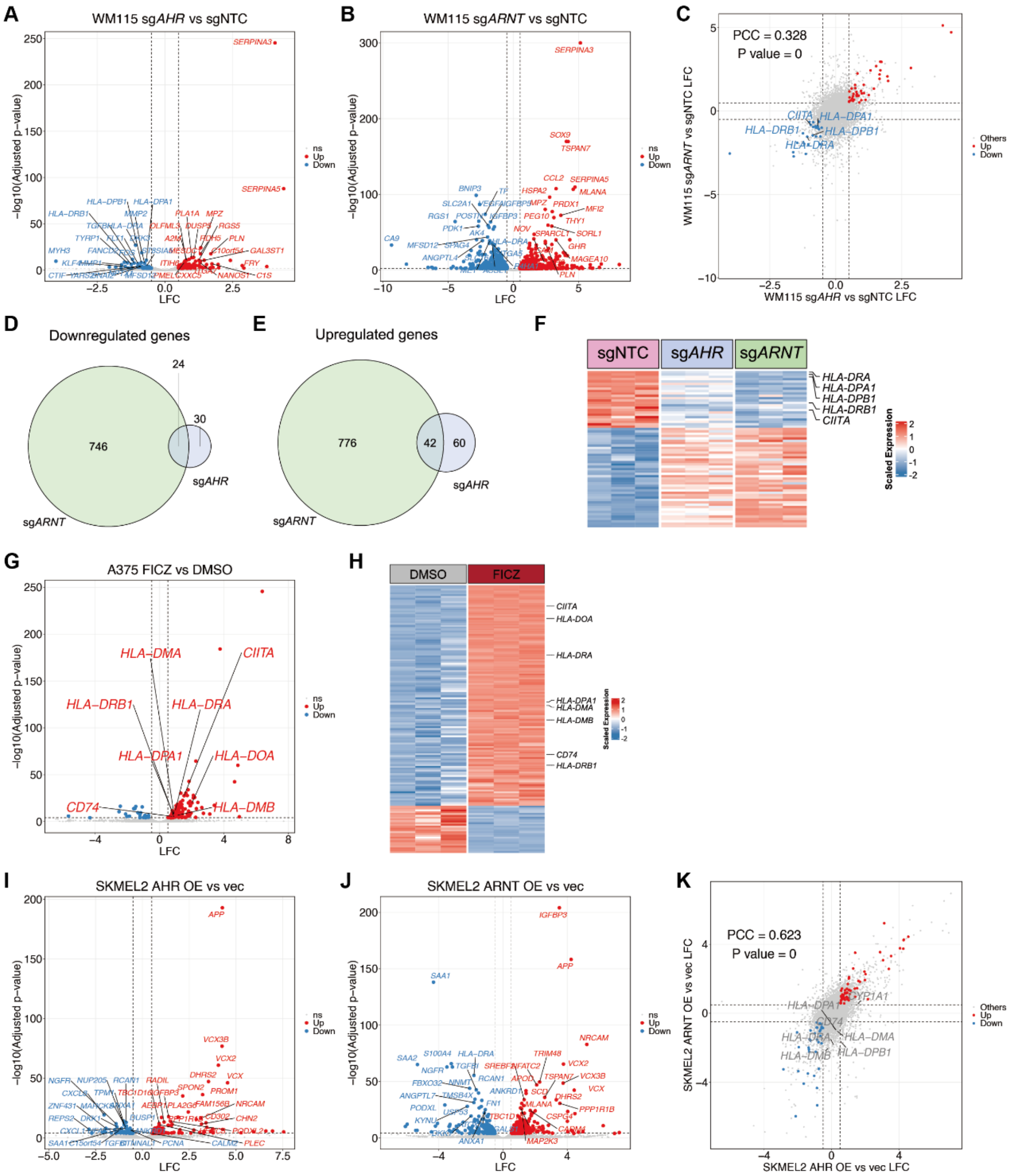

Supplementary Figure 5. AHR signaling modulates HLA-II expression via transcriptional regulation of *CIITA*.

(**A-B**) Scatterplots showing differentially expressed genes in WM115 cells with CRSIPR-mediated knockouts of *AHR* (**A**) or *ARNT* (**B**) compared to NTC cells. Significantly downregulated (blue) and upregulated (red) genes are defined by adjusted $p < 0.01$ and $|\log_2$ fold change$| > 0.5$. The top 20 differentially expressed genes are indicated.

(**C**) Correlation analysis of gene expression changes between *AHR* KO and *ARNT* KO WM115 groups. Each point represents a gene; commonly upregulated (red) and downregulated (blue) genes are highlighted, with HLA-II-related genes indicated.

(**D-E**) Venn diagrams showing the overlap of significantly downregulated (**D**) and upregulated genes (**E**) between *AHR* KO and *ARNT* KO WM115 groups.

(**F**) Heatmap showing the common significantly downregulated and upregulated genes in *AHR* KO, *ARNT* KO, and NTC samples of WM115, with HLA-II-related genes indicated.

(**G**) Scatterplot showing differentially expressed genes in A375 cells treated with 1 μM FICZ for 72 h compared to the 0.1% DMSO control group. Significantly downregulated (blue) and upregulated (red) genes are defined by adjusted $p < 0.0001$ and $|\log_2 \text{fold change}| > 0.5$. HLA-II-related genes are indicated.

(**H**) Heatmap of differentially expressed genes in A375 cells with FICZ versus DMSO control, with HLA-II-related genes annotated.

(**I-J**) Scatterplots showing differentially expressed genes in SKMEL2 cells overexpressing AHR (**I**) or ARNT (**J**) compared to vector control cells. Significantly downregulated (blue) and upregulated (red) genes are defined by adjusted $p < 0.0001$ and $|\log_2 \text{fold change}| > 0.5$. The top 20 differentially expressed genes are indicated.

(**K**) Correlation analysis of gene expression changes between AHR OE and ARNT OE SKMEL2 groups. Each point represents a gene; commonly upregulated (red) and downregulated (blue) genes are highlighted. *CYP1A1* and HLA-II-related genes that are significant in either group are indicated.

Statistical analysis by t-test (**C-K**). PCC, Pearson correlation coefficient; vec, empty vector control.

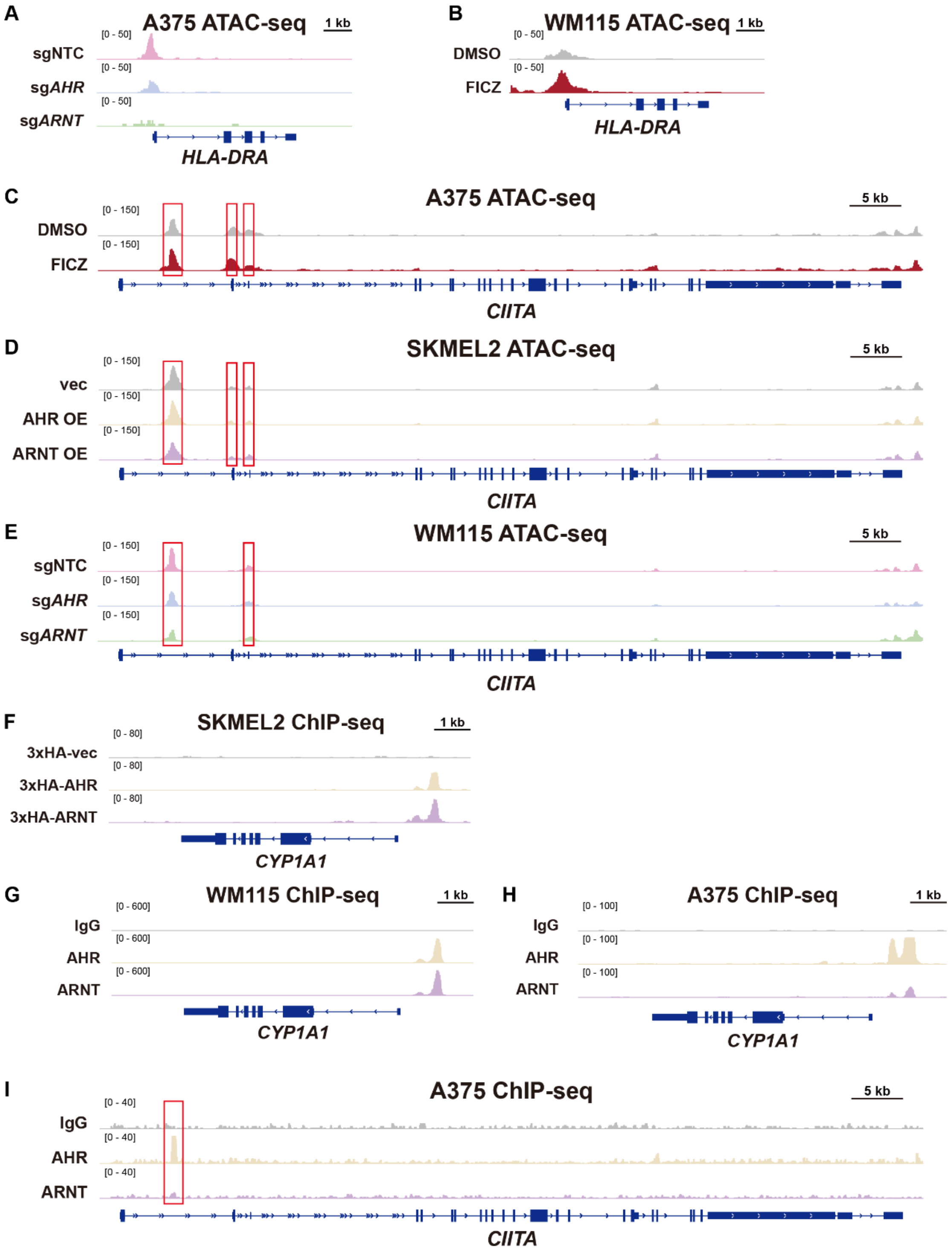

Supplementary Figure 6. AHR-ARNT complex binds *CIITA* promoter II to drive HLA-II transcription.

(**A-B**) ATAC-seq tracks showing chromatin accessibility at the *HLA-DRA* locus in A375 cells with sgNTC, sg*AHR*, or sg*ARNT* (**A**) and in WM115 cells treated with 1 μM FICZ or 0.1% DMSO for 72 h (**B**).

(**C-E**) ATAC-seq tracks showing chromatin accessibility at the *CIITA* locus in A375 cells treated with 1 μM FICZ or 0.1% DMSO for 72 h (**C**), in SKMEL2 cells expressing empty vector control, AHR overexpression, ARNT overexpression constructs (**D**), and in WM115 cells with sgNTC, sg*AHR*, or sg*ARNT* (**E**).

(**F-H**) ChIP-seq tracks showing AHR or ARNT binding at the *CYP1A1* locus in SKMEL2 cells expressing empty vector control, 3×HA-AHR overexpression, or 3×HA-ARNT overexpression constructs, immunoprecipitated with anti-HA antibody (clone 1F5C6) (**F**) and in WM115 cells treated with 1 μM FICZ for 72 h and in A375 cells, immunoprecipitated with IgG control (clone DA1E), anti-AHR (clone D5S6H), or anti-ARNT (clone D28F3) antibodies (**G-H**).

(**I**) ChIP-seq tracks showing AHR or ARNT binding at the *CIITA* locus in A375 cells, immunoprecipitated with IgG control (clone DA1E), anti-AHR (clone D5S6H), or anti-ARNT (clone D28F3) antibodies (**G-H**).

vec, empty vector control.

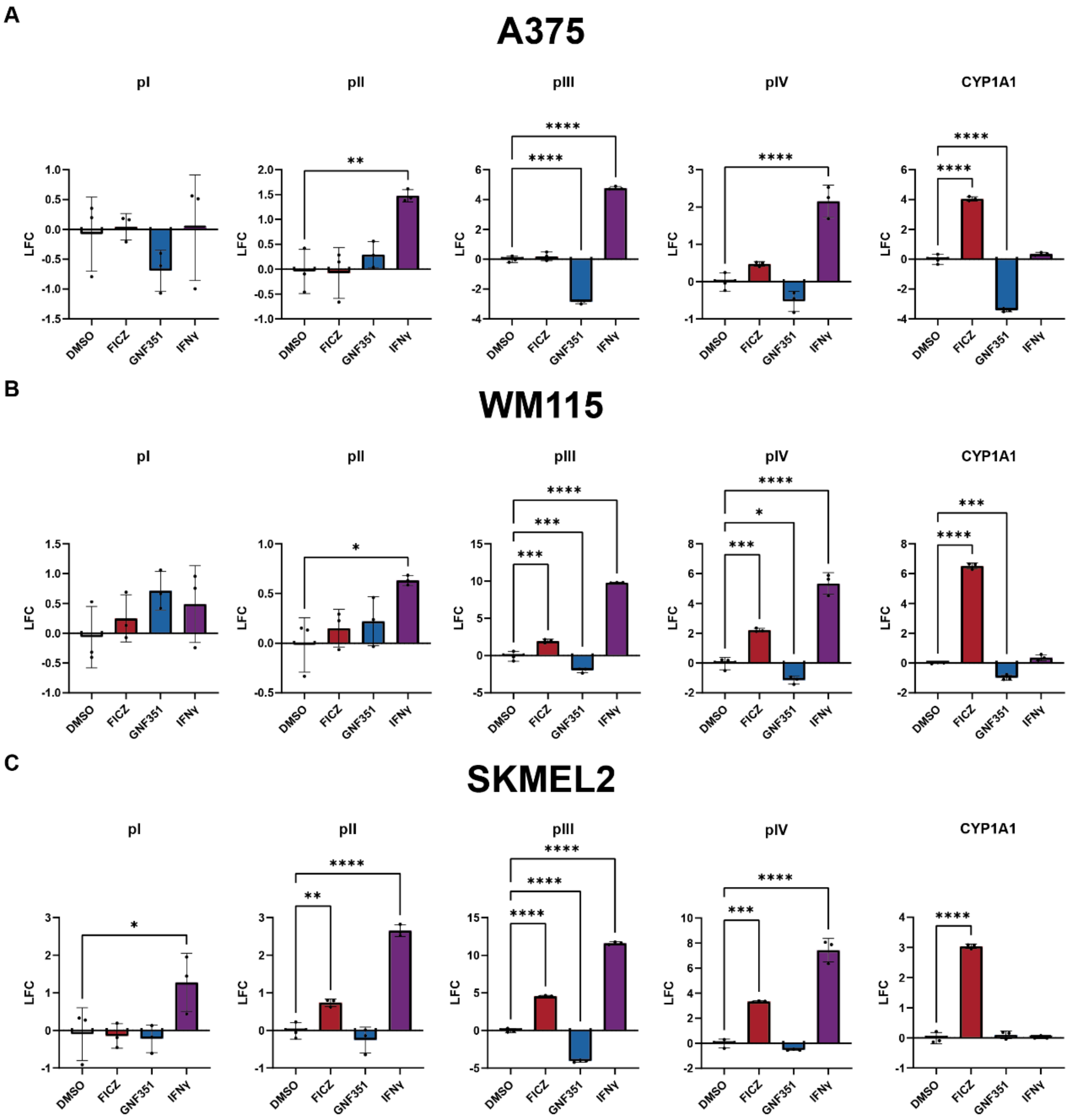

Supplementary Figure 7. AHR-ARNT activation induces transcription of type II, III, and IV *CIITA* mRNA isoforms.

(**A-C**) RT-qPCR analysis of isoform-specific *CIITA* transcripts and *CYP1A1* in A375 (**A**), WM115 (**B**), and SKMEL2 (**C**) cells following 72-hour treatment with 1 μM FICZ (AHR agonist), 2.5 μM GNF351 (AHR antagonist), 100 ng/mL IFN-γ, or 0.1% DMSO as vehicle control. Gene expression levels were normalized to *GAPDH* and are presented as log2 fold change (LFC) relative to the DMSO control group.

Data are represented as mean ± SD (**A-C**). Each dot represents one technical replicate from triplicate wells (**A-C**). Statistical analysis by one-way ANOVA (**A-C**); *$p < 0.05$, **$p < 0.01$, ***$p < 0.001$, ****$p < 0.0001$.
